# *Agromyces chromiiresistens* sp. nov., *Novosphingobium album* sp. nov., *Sphingobium arseniciresistens* sp. nov., *Sphingomonas pollutisoli* sp. nov., and *Salinibacterium metalliresistens* sp. nov.: five new members of *Microbacteriaceae* and *Sphingomonadaceae* from polluted soil

**DOI:** 10.3389/fmicb.2023.1289110

**Published:** 2023-11-28

**Authors:** Ze-Shen Liu, Ke-Huan Wang, Man Cai, Mei-Ling Yang, Xiao-Kang Wang, Hong-Lin Ma, Yi-Han Yuan, Lin-Huan Wu, De-Feng Li, Shuang-Jiang Liu

**Affiliations:** ^1^State Key Laboratory of Microbial Resources, Institute of Microbiology, Chinese Academy of Sciences, Beijing, China; ^2^Institute of Microbiology, University of Chinese Academy of Sciences, Beijing, China; ^3^Institute of Microbiology, Chinese Academy of Sciences, Beijing, China; ^4^School of Life Science, Hebei University, Baoding, China; ^5^State Key Laboratory of Microbial Biotechnology, Shandong University, Qingdao, China

**Keywords:** *Microbacteriaceae*, *Sphingomonadaceae*, *Agromyces*, *Salinibacterium*, *Sphingomonas*, *Sphingobium*, *Novosphingobium*, polluted soil environment

## Abstract

There are many unidentified microbes in polluted soil needing to be explored and nominated to benefit the study of microbial ecology. In this study, a taxonomic research was carried out on five bacterial strains which were isolated and cultivated from polycyclic aromatic hydrocarbons, and heavy metals polluted soil of an abandoned coking plant. Phylogenetical analysis showed that they belonged to the phyla *Proteobacteria* and *Actinobacteria*, and their 16S rRNA gene sequence identities were lower than 98.5% to any known and validly nominated bacterial species, suggesting that they were potentially representing new species. Using polyphasic taxonomic approaches, the five strains were classified as new species of the families *Microbacteriaceae* and *Sphingomonadaceae*. Genome sizes of the five strains ranged from 3.07 to 6.60 Mb, with overall DNA G+C contents of 63.57–71.22 mol%. The five strains had average nucleotide identity of 72.38–87.38% and digital DNA-DNA hybridization of 14.0–34.2% comparing with their closely related type strains, which were all below the thresholds for species delineation, supporting these five strains as novel species. Based on the phylogenetic, phylogenomic, and phenotypic characterizations, the five novel species are proposed as *Agromyces chromiiresistens* (type strain H3Y2-19a^T^ = CGMCC 1.61332^T^), *Salinibacterium metalliresistens* (type strain H3M29-4^T^ = CGMCC 1.61335T), *Novosphingobium album* (type strain H3SJ31-1^T^ = CGMCC 1.61329^T^), *Sphingomonas pollutisoli* (type strain H39-1-10^T^ = CGMCC 1.61325^T^), and *Sphingobium arseniciresistens* (type strain H39-3-25^T^ = CGMCC 1.61326^T^). Comparative genome analysis revealed that the species of the family *Sphingomonadaceae* represented by H39-1-10^T^, H39-3-25^T^, and H3SJ31-1^T^ possessed more functional protein-coding genes for the degradation of aromatic pollutants than the species of the family *Microbacteriaceae* represented by H3Y2-19a^T^ and H3M29-4^T^. Furthermore, their capacities of resisting heavy metals and metabolizing aromatic compounds were investigated. The results indicated that strains H3Y2-19a^T^ and H39-3-25^T^ were robustly resistant to chromate (VI) and/or arsenite (III). Strains H39-1-10^T^ and H39-3-25^T^ grew on aromatic compounds, including naphthalene, as carbon sources even in the presence of chromate (VI) and arsenite (III). These features reflected their adaptation to the polluted soil environment.

## 1 Introduction

*Microbacteriaceae* and *Sphingomonadaceae* are families belonging to *Actinobacteria* and *Proteobacteria*, respectively, and have been frequently detected in diverse environments, including polluted environments with polycyclic aromatic hydrocarbons (PAHs) and heavy metals. The species of the family *Microbacteriaceae*, for example, *Agromyces* and *Salinibacterium*, were repeatedly detected in polluted environments (Papale et al., 2017; Parab and Phadke, 2020; Huang et al., 2022; Sazykina et al., 2022). *Agromyces* and *Salinibacterium* were also detected in habitats such as caves, marine environments, soil, sediments, and rhizosphere (Jurado et al., 2005; Hamada et al., 2014; Wang et al., 2016; Lu et al., 2023). At the time of writing the article, *Agromyces* harbors 42 validly nominated species and 4 subspecies (https://lpsn.dsmz.de/genus/agromyces), with *Agromyces ramosus* DSM 43045^T^ as the type strain (Gledhill and Casida, 1969). *Salinibacterium* harbors three validly nominated species (https://lpsn.dsmz.de/genus/salinibacterium), with *Salinibacterium amurskyense* KMM 3673^T^ as the type strain (Han et al., 2003).

The family *Sphingomonadaceae* (https://lpsn.dsmz.de/family/sphingomonadaceae) is ubiquitous in environments, and members of this family are active degraders of pollutants, including PAHs (Brito et al., 2006; Shokrollahzadeh et al., 2008; Waigi et al., 2015). Its type genus *Sphingomonas* was first proposed in 1990 and then split into four taxa, namely, *Sphingomonas sensu stricto* (*Spm*), *Sphingobium* (*Spb*), *Novosphingobium*, and *Sphingopyxis* (Takeuchi et al., 2001). *Sphingomonadaceae* species share the unique cellular component of sphingoglycolipids (Singh et al., 2015). At the time of writing the article, *Novosphingobium* harbors 58 validly published and correct species names (https://lpsn.dsmz.de/genus/novosphingobium), with *Novosphingobium capsulatum* as the type species. *Sphingobium* harbors 44 validly nominated species, with *Sphingobium yanoikuyae* as the type species (https://lpsn.dsmz.de/genus/sphingobium). *Sphingomonas* harbors 152 validly published and correct species names, with *Sphingomonas paucimobilis* (Yabuuchi et al., 1990) as the type species (https://lpsn.dsmz.de/genus/sphingomonas).

Many soil microbes remain uncultivated (Daniel, 2005; Lok, 2015), and microbes dwelling in polluted soil may adapt themselves by evolution for resistance or even assimilation of chemical pollutants such as PAHs and heavy metals for energy or carbon sources (Gou et al., 2020; Thakur et al., 2022). We previously explored the microbial diversity of polluted sites of the coking plant and found that the microbial taxa responded differently to PAHs and heavy metals (Yang et al., 2022). This communication reports the isolation and genotypic and phenotypic characterization of five bacterial strains, namely, H3Y2-19a^T^, H3M29-4^T^, H39-1-10^T^, H39-3-25^T^, and H3SJ31-1^T^, from the polluted soil samples. With polyphasic taxonomic approaches, those strains were identified and classified into five novel species pertaining to the genera *Agromyces* and *Salinibacterium* of the family *Microbacteriaceae* and genera *Novosphingobium, Sphingobium*, and *Sphingomonas* of the family *Sphingomonadaceae*.

## 2 Materials and methods

### 2.1 Sample collection

Soil samples were collected from an abandoned coking plant that operated for 60 years in Hangzhou City, Zhejiang Province, China. The sampling site is located near the center of the plant area and a mass of black and sticky contaminants was observed from the soil section of the sampling site. Five different soil types from the upper-30 cm layer were mixed to obtain one composite sample. The samples show a visibly black appearance and a crude oil smell. The climate of Hangzhou is a subtropical monsoon with four distinct seasons, which is warm and humid. Sampling was conducted in December 2020, when it was wintertime with the local temperature of 5–9°C. After collecting the soil samples, they were immediately stored in sterile PE bags and transported to the laboratory. The soil samples were analyzed for PAHs and heavy metals in the laboratory using HPLC and ICP-OES, and the results showed that the soil was severely polluted by PAHs with concentrations as high as 12,558.06±611.19 mg/kg and was slightly polluted by heavy metals (Yang et al., 2022).

### 2.2 Culture media, isolation, and cultivation conditions

Soils were suspended in a sterile phosphate buffer solution and shaken for 2 h to prepare the cell suspension. The cell suspensions after being diluted were spread onto agar plates and cultured in different conditions. To make M1 medium, the concentrations of K_2_HPO_4_ and MgSO_4_.7H_2_O were in accordance with that of R2A (Lee and Whang, 2020), while other components were diluted five times. The final pH was adjusted to 7.2 by adding K_2_HPO_4_ or KH_2_PO_4_. The medium was autoclaved for 30 min at 105°C. To make M2 medium, 200 μl of a PAH matrix was spread on the mineral salt medium (MSM) agar plates (Samsu et al., 2020). A mixed solution of benz(a)anthracene and benzo(a)pyrene dissolved in acetone (1 g/L, respectively) was used as the PAH matrix. The matrix was sterilized by a 0.22-μm sterile filter. Before spreading the cell suspension, the acetone was volatilized. To make M3 medium, the soil extract solution was added into M1 with 10% dosage and then autoclaved for 30 min at 105°C. The soil extract solution was prepared by the addition of 5.0 g soil into 150 ml distilled water, rotatory shaking at 150 rpm for 30 min, and then centrifuged to obtain supernatant. The agar plates were cultivated at room temperature (20–25°C) or 30°C. Colonies were picked up following their gradual appearance. Colonies were re-streaked on M1 agar plates until obtaining the pure strain.

### 2.3 Heavy metal resistance and aromatic compound metabolism

The five bacterial strains were cultured in R2A medium to prepare the seed cultures for heavy metal resistance and aromatic compound metabolism experiments. The seed cultures were washed twice using sterile PBS. After sterilization, the R2A medium was supplemented with 0.5, 2.0, 5.0, and 10.0 mM sterilized sodium arsenite or 5, 20, 50, and 100 mg/L sterilized potassium dichromate. To each well of 96 microwell plates, 150 μl heavy metal-containing R2A medium and inoculated 5 μl washed cells was added. The 96 microwell plates were cultured at 30°C for 13 days, and OD_600_ was recorded at intervals using a microplate reader. The maximum increased OD_600_ from the initial values of each well was picked out to determine the growth of bacteria. In aromatic compound metabolism experiments, 100 mg/L of 2,5-dihydroxybenzoic acid, 4-hydroxybenzoic acid, protocatechuic acid, salicylic acid, phthalic acid, benzoic acid, naphthalene, or phenanthrene were added into MSM medium, respectively. Each well of 96 microwell plates was infused with 150 μl MSM medium and inoculated with 5 μl washed cells. The 96 microwell plates were cultured at 30°C for 30 days. OD_600_ was recorded at intervals using a microplate reader. All the tests were carried out in duplicate.

To examine the PAH-degrading ability of isolates, phenanthrene (200 mg/L) or naphthalene (200 mg/L) accompanied with or without heavy metals was added into 5 ml MSM medium, respectively. The heavy metal was 0.5 mM sodium arsenite or 5 mg/L of potassium dichromate. PAH degradation was tested in the presence of arsenite or dichromate. Bacterial cells were washed and inoculated into the medium at 2% (v/v) and incubated for 3 days for naphthalene degradation and 10 days for phenanthrene degradation. At the end of cultivation, the residual PAHs were extracted using dichloromethane and detected by HPLC (Sakshi et al., 2022; Yang et al., 2022). The tubes without inoculation were set as control. The reduction of PAH content compared with control was calculated to determine the degradation percent. All the degradation tests were carried out in triplicates.

### 2.4 Morphology observation and chemotaxonomic determinations

Morphology of bacterial colony was observed after aerobically culturing on R2A agar plates at 30°C for 2–5 days. The single-cell morphology was observed by transmission electron microscopy (JEM-1400, Joel). The metabolic profiling of the carbon source was investigated using Biolog^TM^ GEN III microplate systems according to the manufacturer protocol, which contained 71 different carbon source utilization assays and 23 chemical sensitivity assays. To make significative comparisons, the cell biomass for fatty acid identification of each bacterial strain was cultured in the corresponding medium reported for their reference type strains. H39-3-25^T^ was cultured in R2A medium. H3M29-4^T^ was cultured in a marine agar medium (Han et al., 2003). H39-1-10^T^ was cultured in an NA medium (Singh et al., 2015). H3SJ31-1^T^ and H3Y2-19a^T^ were cultured in TSA medium (Gupta et al., 2009). Cells were harvested during the exponential growth phase. Cellular fatty acids were then extracted, methylated according to the standard MIDI protocol (Sherlock Microbial Identification System, version 6.0), and analyzed with a gas chromatograph (HP 6890 Series GC System, Agilent) (Sasser, 1990). Polar lipids were separated by two-dimensional thin-layer chromatography on silica TLC plates (10 × 10cm; Merck), for which chloroform/methanol/water (65:25:4, in volume) and chloroform/methanol/acetic acid/water (80:12:15:4, in volume) were used as the first- and second-dimensional spreading agents, respectively (Minnikin et al., 1984). After separation, the polar lipids were detected by spraying reagents as follows: 10% ethanolic molybdophosphoric acid for total lipids, 0.4% ninhydrin solution in butanol for aminolipids, 1.3% molybdenum blue spray reagent for phospholipids, and 0.5% α-naphthol sulfuric acid reagent for glycolipids.

### 2.5 16S rRNA gene sequencing and phylogenetic analysis

The complete 16S rRNA genes were amplified using the universal primers 27F (5′- AGAGTTTGATCCTGGCTCAG−3′) and 1492R (5′- GGTTACCTTGTTACGACTT-3′) (Soergel et al., 2012). The gene similarities to previously reported type strains were determined using the EzBioCloud server (Yoon et al., 2017). The 16S rRNA gene sequences of type strains were downloaded from the EzBioCloud server and aligned using CLUSTAL W (Thompson et al., 1994). Phylogenetic trees were constructed using MEGA 11 software based on the neighbor-joining (NJ) method according to Kimura's two-parameter model (Kimura, 1980), maximum-likelihood (ML) method based on the Tamura–Nei model (Tamura and Nei, 1993), and maximum-parsimony (MP) algorithms based on the Subtree-Pruning-Regrafting (SPR) search method (Fitch, 1971). The statistical reliability of these trees was conducted using bootstrap analysis with 1,000 replications (Felsenstein, 1985).

### 2.6 Genome sequencing and analysis

Genomic DNA was extracted using commercial TIANamp bacteria DNA kits and sequenced on an Illumina Hiseq X-ten platform. After quality control, the reads were assembled with multiple assemblers to obtain the best assembly, and the predicted genes were annotated using DIAMOND software by referring to databases (including KEGG, COG, NR, SwissProt, and Pfam), as described in the Global Catalog of Type Strain (gcType) Platform Manual v2 (https://gctype.wdcm.org/manual.jsp#detail). The RAST annotation engine (http://rast.nmpdr.org/) was also used to annotate genes. To assess the genome-based phylogeny, whole-genome-based phylogenomic trees were constructed using the up-to-date bacterial core genes (UBCGs) set pipeline (www.ezbiocloud.net/tools/ubcg) (Na et al., 2018). Average nucleotide identity (ANI) values and digital DNA–DNA hybridization (dDDH) were calculated using the ANI calculator (https://www.ezbiocloud.net/tools/orthoani) and Genome-to-Genome Distance Calculator 3.0 (GGDC; https://ggdc.dsmz.de/ggdc.php#) along with UPGMA dendrogram (unweighted pair group method with arithmetic mean) (Meier-Kolthoff et al., 2013; Lee et al., 2016). The DNA G+C contents were also determined by GGDC 3.0. Genome analysis by Check M showed that the genomes of all strains were not contaminated (Parks et al., 2015).

For comparative genome and genomic synteny analyses, the nearest phylogenomic relatives to the five strains with available genome sequences were chosen, and their genomes were retrieved from the NCBI database. The coding sequences of the genomes were predicted using GeneMarkS and Glimmer 3.02 software (http://ccb.jhu.edu/software/glimmer/index.shtml). The comparison of orthologous gene clusters was carried out using OrthoVenn2 software (https://orthovenn2.bioin fotoolkits.net/home). The genomic synteny analyses were carried out using MUMmer software by Promer way and visualized in a dot plot (Kurtz et al., 2004). Syntenic blocks of DNA sequence were analyzed using Mauve software (Darling et al., 2004). The KEGG pathways were predicted through the KEGG automatic annotation server (KAAS), and the richness of functional genes based on KEGG pathway level 3 was visualized in a heatmap. The distribution of homologous gene clusters was displayed using the Bioplot module of Chiplot (https://www.chiplot.online/).

### 2.7 Culture preservation

All the assigned type strains in this study were deposited at the China General Microbiological Culture Collection Center (CGMCC). The accession numbers of these strains are provided in the section of the species description.

## 3 Results and discussion

### 3.1 Five strains representing potentially novel species and their genomes

**Figure 1:**
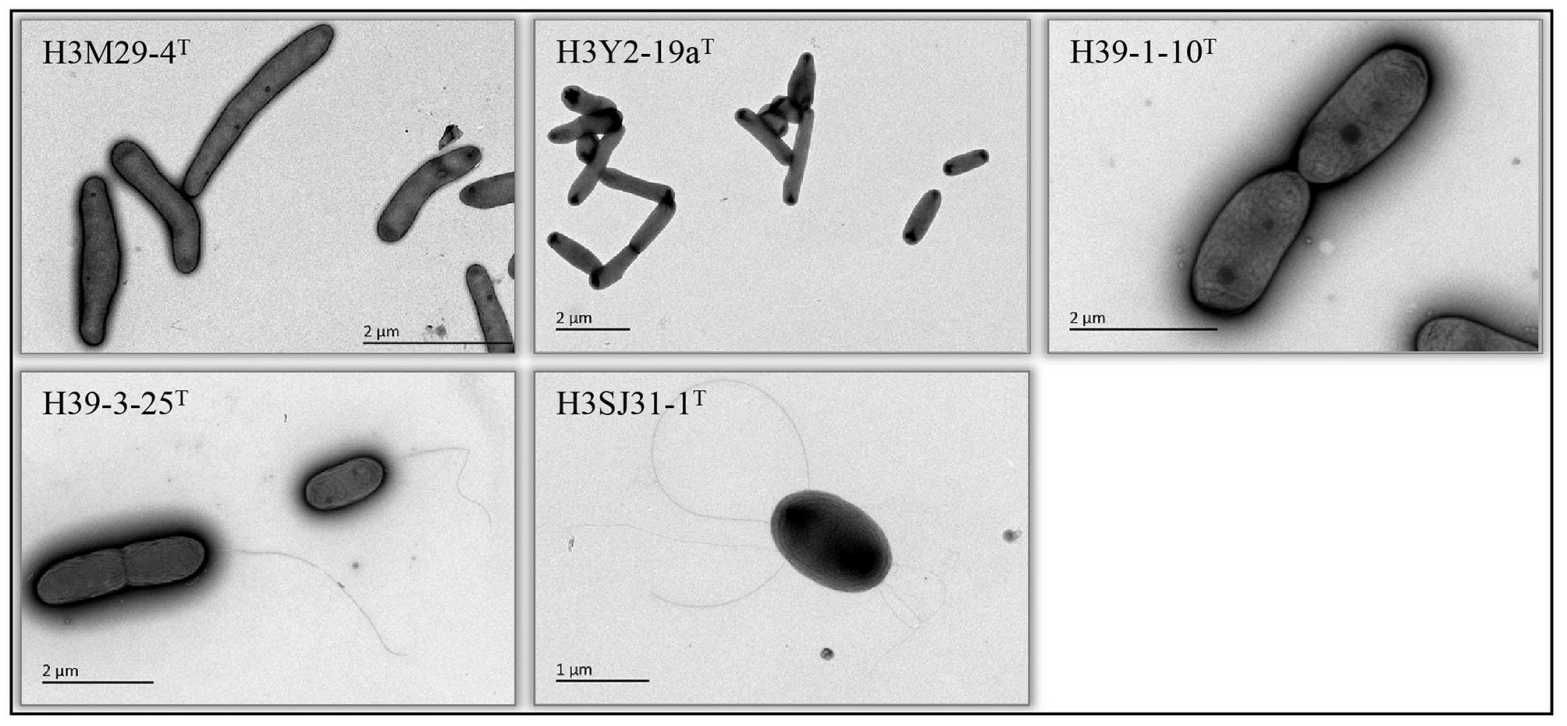
Cellular morphology (transmission electron microscopy) of the five bacteria isolated from polluted soil. The names of each bacterium and the scale bars of cellular size are shown in the pictures.

Five bacterial strains, namely, H3Y2-19a^T^, H3M29-4^T^, H39-1-10^T^, H39-3-25^T^, and H3SJ31-1^T^, that were phylogenetically related to previously known and validly nominated species of the families *Microbacteriaceae* and *Sphingomonadaceae* (16S rRNA gene sequence identities ranging from 97.22% to 98.36%) were obtained. Specifically, H3Y2-19a^T^ was close to *Agromyces marinus* H23-8^T^ (97.22%), H3M29-4^T^ was close to *Salinibacterium hongtaonis* 194^T^ (97.51%), H39-1-10^T^ was close to *Sphingomonas panacis* DCY99^T^ (98.36%), H39-3-25^T^ was close to *Sphingobium aquiterrae* SKLS-A10^T^ (97.95%), and H3SJ31-1^T^ was close to *Novosphingobium mathurense* SM117^T^ (97.53%). Strains H3Y2-19a^T^ and H3M29-4^T^ were obtained from M1 agar plates after cultivating at 30°C and room temperature, respectively. Strains H39-1-10^T^ and H39-3-25^T^ were obtained from M2 agar plates after cultivating both at room temperature. H3SJ31-1^T^ was obtained from M3 agar plates after cultivating at room temperature. All strains grew on R2A agar plates and generated visible colonies in 2–3 days. The cells of H3M29-4^T^, H39-3-25^T^, H39-1-10^T^, and H3Y2-19a^T^ were rod, and H3SJ31-1^T^ was ovoid-shaped. Cells of H3SJ31-1^T^ and H39-3-25^T^ showed polar flagella (Figure 1).

The genomes of the five strains were sequenced and annotated. The genome sizes and annotated gene numbers are provided in Tables 1, 2. Interpretation of genome annotation and their physiology such as carbon source assimilation, resistances to heavy metals and chemicals, and biodegradation of aromatic compounds are described in the following paragraphs. The genomic DNA G+C molar contents of H3Y2-19a^T^, H3M29-4^T^, H39-1-10^T^, H39-3-25^T^, and H3SJ31-1^T^ were 71.22, 69.07, 66.15, 63.57, and 66.16 mol%, respectively.

**Table 1:** Phenotypic, chemotaxonomic, and genomic features of two novel species of *Microbacteriaceae*, and differentiation to their closely related species.

**Strains**	**1**	**2**	**3**	**4**	**5**	**6**
**Cellular properties**						
Morphology	Rod-shaped	Diphtheroid and rod-shaped	Coccoid to rod-shaped	Rod-shaped	Rod-shaped	Rod-shaped
Appendage	no	no	no	no	/	/
Size (μm)	1.26–2.58 L; 0.39-0.57 W	0.3–0.5W	0.4–0.6 W	1.73–2.86 L; 0.30–0.42 W	1.2–2.0L; 0.5–0.8 W	/
**Colony**	White to fluorescent yellow, circular, convex	Circular, convex, smooth, cream	Pale yellow, circular, transparent, smooth	Pale yellow, smooth, circular, translucent	Pale yellow, circular, non-transparent	Non-motile, aerobic, non-spore-forming, irregular
**Genome features**						
Genome size (Mbp)	4.09	3.73	/	3.07	2.81	2.78
Gene number	3,665	3,382	/	3,022	2,709	2,678
DNA G+C content (mol%)	71.22	70.80	72.50	69.07	64.10	61.90
Identity of 16S rRNA gene (%)		97.58	97.22		97.51	96.94
ANI (%)		84.07	/		72.38	72.63
dDDH (%)		27.1	/		14.0	14.0
**Major fatty acids**	iso-C_15:0_, iso-C_16:0_, anteiso-C_15:0_, anteiso-C_17:0_	iso-C_15:0_, iso-C_16:0_, anteiso-C_15:0_, anteiso-C_17:0_	iso-C_16:0_, anteiso-C_15:0_, anteiso-C_17:0_	iso-C_16:0_, anteiso-C_15:0_, anteiso-C_17:0_	iso-C_16:0_, anteiso-C_15:0_, anteiso-C_17:0_	iso-C_14:0_, anteiso-C_15:0_
**Major polar lipids**	DPG, PG, GL, L	DPG, PG, GL, PL	DPG, PG, GL	DPG, PG, GL, L	DPG, PG, GL	DPG, PG

1, H3Y2-19a^T^; 2, *Agromyces italicus* DSM 16388^T^ (Jurado et al., 2005); 3, *Agromyces marinus* H23-8^T^ (Hamada et al., 2014); 4, H3M29-4^T^; 5, *Salinibacterium hongtaonis* 194^T^ (Li et al., 2019); 6, *Salinibacterium amurskyense* KMM 3673^T^ (Han et al., 2003). /, not reported. Identity of the 16S rRNA gene, and ANI and dDDH refer to the value of comparing the 16S rRNA gene sequence or genome sequence with their respective closely related type strains. DPG, diphosphatidylglycerol; PG, phosphatidylglycerol; PL, unknown phospholipid; L, unknown lipid; GL, unknown glycolipid.

**Table 2:** Phenotypic, chemotaxonomic, and genomic features of three novel species of *Sphingomonadaceae*, and differentiation to their closely related species.

**Strains**	**1**	**2**	**3**	**4**	**5**	**6**
**Cellular properties**						
Morphology	Rod-shaped	Rod-shaped	Rod-shaped	Rod-shaped	Ovoid-shaped	Non-sporulating rod-shaped
Appendage	No	Flagellum	Polar flagellum	Polar flagellum	Polar flagellum	Polar flagellum
Size (μm)	2.14–3.33 L; 0.95-1.18 W	/	1.47–2.62 L; 0.68–0.81 W	0.7–1.1 L; 0.4 W	0.89–1.15 L; 0.73–0.90 W	1.4 L; 0.8 W
**Colony**	Circular, milk white, non-transparent	Circular, convex, smooth, non-transparent, yellow	Pale yellow, viscous, non-transparent	Pale yellow, round, smooth	Milk white, non-transparent	Yellow, smooth, circular, convex
**Genome features**						
Genome size (Mbp)	5.55	5.32	6.60	/	5.03	4.84
Gene number	5,234	4,889	6,135	/	4,763	4,490
DNA G+C content (mol%)	66.15	64.40	63.57	65.90	66.16	63.30
Identity of 16S rRNA gene (%)		98.36		97.95		97.53
ANI (%)		87.38		/		76.91
dDDH (%)		34.2		/		20.7
**Major fatty acids**	C_16:0_, C_14:0_ 2-OH, summed feature 8	C_16:0_, C_14:0_ 2-OH, summed feature 8	C_16:0_, C_14:0_ 2-OH, summed feature 8	C_14:0_ 2-OH, summed feature 8	C_16:0_, summed feature 8	Summed feature 8, summed feature 3
**Major polar lipids**	DPG, PE, PG, PC, SGL, APL, GL, and L	DPG, PE, PG, PC, and SGL	DPG, PE, PG, PME, SGL, and PL	DPG and SGL	DPG, PE, PG, PC, PME, PDE, SGL, and L	DPG, PE, PG, PC, PME, PDE, SGL, GPL, GL, and PL

1, H39-1-10^T^; 2, *Sphingomonas panacis* DCY99^T^ (Singh et al., 2015); 3, H39-3-25^T^; 4, *Sphingobium aquiterrae* SKLS-A10^T^ (Revesz et al., 2018); 5, H3SJ31-1^T^; 6, *Novosphingobium mathurense* SM117^T^ (Gupta et al., 2009). /, not reported. Identity of the 16S rRNA gene, and ANI and dDDH refer to the value of comparing the 16S rRNA gene sequence or genome sequence with their respective closely related type strains. DPG, diphosphatidylglycerol; PG, phosphatidylglycerol; PE, phosphatidylethanolamine; PC, phosphatidylcholine; PME, phosphatidyl monomethylethanolamine; PDE, phosphatidyl dimethylethanolamine; SGL, sphingoglycolipid; APL, unknown aminophospholipid; PL, unknown phospholipid; L, unknown lipid; GL, unknown glycolipid; GPL, unknown glycophospholipid. Summed features represent the integration of two or three fatty acids that cannot be differentiated by the MIDI system. Summed feature 3 included C_16:1_ ω7c and/or C_16:1_ ω6c; summed feature 8 included C_18:1_ ω7c and/or C_18:1_ ω6c.

### 3.2 Assimilation of carbon sources and response to chemical sensitivity assays

**Figure 2:**
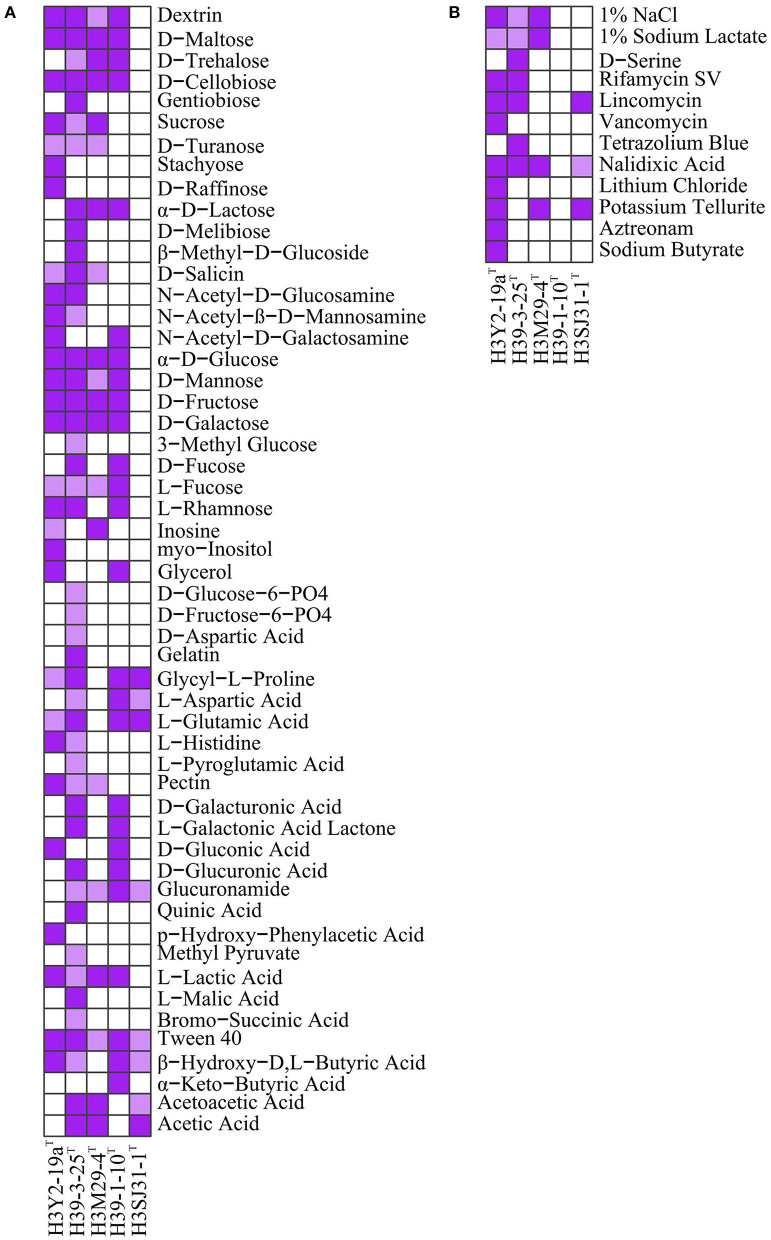
Assimilation of carbon sources **(A)** and responses to chemical sensitivity assays **(B)** of the five strains on Biolog^TM^ GEN III microplates. Deep purple indicates strong positive, light purple indicates weak positive, and white indicates negative. All strains had no growth on the carbon sources of N-acetyl neuraminic acid, _D_-sorbitol, _D_-mannitol, _D_-arabitol, _D_-serine, _L_-alanine, _L_-arginine, _L_-serine, mucic acid, _D_-saccharic acid, _D_-lactic acid methyl ester, citric acid, α-keto-glutaric acid, _D_-malic acid, γ-amino-butryric acid, α-hydroxy-butyric acid, propionic acid, and formic acid and was negative to 4% NaCl, 8% NaCl, fusidic acid, troleandomycin, minocycline, guanidine HCl, niaproof 4, tetrazolium violet, and sodium bromate at pH 5.0. All strains showed positive to Tween 40 and pH 6.0.

The results of 71 carbon source assimilation and 23 chemical sensitivity assays on Biolog^TM^ GEN III microplates are shown in Figure 2. H3Y2-19a^T^, H39-3-25^T^, H3M29-4^T^, and H39-1-10^T^ showed similar metabolic profiles to monosaccharides (α-_D_-glucose, _D_-mannose, _D_-fructose, _D_-galactose, _L_-fucose, and _L_-rhamnose), disaccharides (_D_-maltose, _D_-trehalose, _D_-cellobiose, sucrose, and _D_-turanose), and oligosaccharides (dextrin). Among the five bacteria, H39-3-25^T^ exhibited the most potent metabolic ability of carbon sources and utilized a total of 44 carbon sources. H3Y2-19a^T^, H3M29-4^T^, and H39-1-10^T^ utilized 29, 20, and 26 carbon sources, respectively. By contrast, H3SJ31-1^T^ could only utilize eight carbon sources. Particularly, H3SJ31-1^T^ could not metabolize saccharides. This was in accordance with the genome annotation results that H3Y2-19a^T^, H39-3-25^T^, H3M29-4^T^, and H39-1-10^T^ possessed 71, 43, 33, and 76 genes for metabolizing monosaccharides, disaccharides, and oligosaccharides, respectively, while H3SJ31-1^T^ possessed only 7 related genes, as shown in Supplementary Table S1.

The overall results from chemical sensitivity assays showed that strains H3M29-4^T^, H39-1-10^T^, and H3SJ31-1^T^ were more sensitive to chemical agents than H3Y2-19a^T^ and H39-3-25^T^. In the 23 chemical agents, H3M29-4^T^ was sensitive to 18 agents, and tolerated pH 6.0, 1% NaCl, 1% sodium lactate, nalidixic acid and potassium tellurite. It did not show growth at the conditions of pH lower than 5.0, and sodium chloride higher than 4%. H3SJ31-1^T^ was sensitive to 19 agents, and tolerated pH 6.0, lincomycin, nalidixic acid and potassium tellurite. It did not show growth at the condition of pH lower than 5.0. H39-1-10^T^ was sensitive to 22 agents. By contrast, H3Y2-19a^T^ showed the strongest tolerance (resisting 11 chemical agents). According to genome annotation, all bacteria had genes for resistance to fluoroquinolones (Supplementary Table S1), and H3Y2-19a^T^, H39-3-25^T^, H3M29-4^T^, and H3SJ31-1^T^ showed growth in nalidixic acid. Genes encoding for β-lactamase (Bonomo, 2017) were annotated in the genomes of H39-1-10^T^, H39-3-25^T^, and H3SJ31-1^T^, but they did not show experimentally resistance to aztreonam. H3Y2-19a^T^ was annotated two genes for encoding multidrug resistance efflux pumps (Alibert et al., 2017) and showed resistance to five antibiotics, namely, rifamycin SV, lincomycin, vancomycin, nalidixic acid, and aztreonam. Unexpectedly, strain H39-1-10^T^ was annotated by nine genes of multidrug resistance efflux pumps, but it did not resist any antibiotics tested with the Biolog^TM^ GEN III microplate.

### 3.3 Cellular fatty acids and polar lipid profiles

Major cellular fatty acids and polar lipids of the five bacteria are summarized in Tables 1, 2, and detailed results are provided in Supplementary Tables S2, S3 and Supplementary Figure S1. Taking 10% as the cutoff value for predominant cellular fatty acids, strain H3Y2-19a^T^ had iso-C_15:0_, iso-C_16:0_, anteiso-C_15:0_, and anteiso-C_17:0_, strain H3M29-4^T^ had iso-C_16:0_, anteiso-C_15:0_, and anteiso-C_17:0_, strains H39-1-10^T^ and H39-3-25^T^ had C_16:0_, C_14:0_ 2-OH, and summed feature 8 (C_18:1_ ω7c and/or C_18:1_ ω6c), and strain H3SJ31-1^T^ had C_16:0_ and summed feature 8 as the major fatty acids. Polar lipid profiles showed that all five bacteria had diphosphatidylglycerol and phosphatidylglycerol but were different in the presence or absence of phosphatidylethanolamine, phosphatidylcholine, sphingoglycolipid, aminophospholipid, phosphatidyl monomethylethanolamine, phosphatidyl dimethylethanolamine, unknown phospholipids, unknown glycolipids, and unknown lipids.

### 3.4 Heavy metal resistance and aromatic compound metabolism

As the five strains were isolated from heavy metals and/or PAH-polluted soil samples, we tested their abilities to resist heavy metals and degrade PAHs. The results showed that strains H39-3-25^T^, H39-1-10^T^, and H3M29-4^T^ also displayed arsenite resistance, and H39-3-25^T^ and H3M29-4^T^ displayed dichromate resistance (Table 3). The strain H3Y2-19a^T^ was the most resistant one and tolerated up to 10 mM of arsenite and 100 mg/L of potassium dichromate. We observed that strain H3SJ31-1^T^ did not show any growth in the presence of arsenite or dichromate. The genome annotations indicated that strain H39-1-10^T^ possessed the gene homologs to *ars*B (Kaur et al., 2011), *chrB*, and *chrR* (Ackerley et al., 2004; He et al., 2018), which played the roles of arsenite and dichromate resistance, respectively (Supplementary Table S4). Surprisingly, we did not identify any homologs to known chromium resistance genes from the most tolerant H3Y2-19a^T^. *Agromyces* was detected as an abundant member in the rhizosphere microbial community and was positively related to Zn and Cd accumulation in plants growing in polluted soils (De Maria et al., 2011). The arsenite and dichromate-resistant strain H3Y2-19a^T^, which was a potential new species of *Agromyces*, would expand its bioresources and would be beneficial for the phytoremediation of heavy metals.

**Table 3:** Characteristics of heavy metal resistance and aromatic compound metabolization of the five novel species.

	**H3Y2-19a^T^**	**H3M29-4^T^**	**H39-1-10^T^**	**H39-3-25^T^**	**H3SJ31-1^T^**
**Sodium arsenite (mM)**					
0.5	++++	++	++++	++++	-
2.0	+	-	-	+++	-
5.0	+	-	-	-	-
10.0	+	-	-	-	-
**Potassium dichromate (mg/L)**					
5	+++	+++	-	++++	-
20	++++	+	-	+++	-
50	++++	-	-	-	-
100	++++	-	-	-	-
**Aromatic compounds**					
Naphthalene	-	-	++++	++	-
Phenanthrene	-	-	-	-	-
2,5-dihydroxybenzoic acid	-	-	++	++	-
*p*-Hydroxybenzoic acid	-	-	+	+	-
Protocatechuic acid	-	-	+	+	-
Salicylic acid	-	-	++	+	-
Phthalic acid	-	-	+	+	-
Benzoic acid	-	-	+	+	-

For heavy metal resistance tests, the bacteria were cultured in R2A medium supplemented with 0.5, 2.0, 5.0, and 10.0 mM sodium arsenite or 5, 20, 50, and 100 mg/L potassium dichromate. For aromatic compound metabolizing tests, the bacteria were cultured in MSM medium supplemented with 100 mg/L of each aromatic compound as the sole carbon source, respectively. Bacteria could be generally regarded as having growth when the OD_600_ value increased by at least 0.1. The OD_600_ value was represented by -, <0.1; +, 0.1–0.2; ++, 0.2–0.3; +++, 0.3–0.4; ++++, ≥0.4.

**Figure 3:**
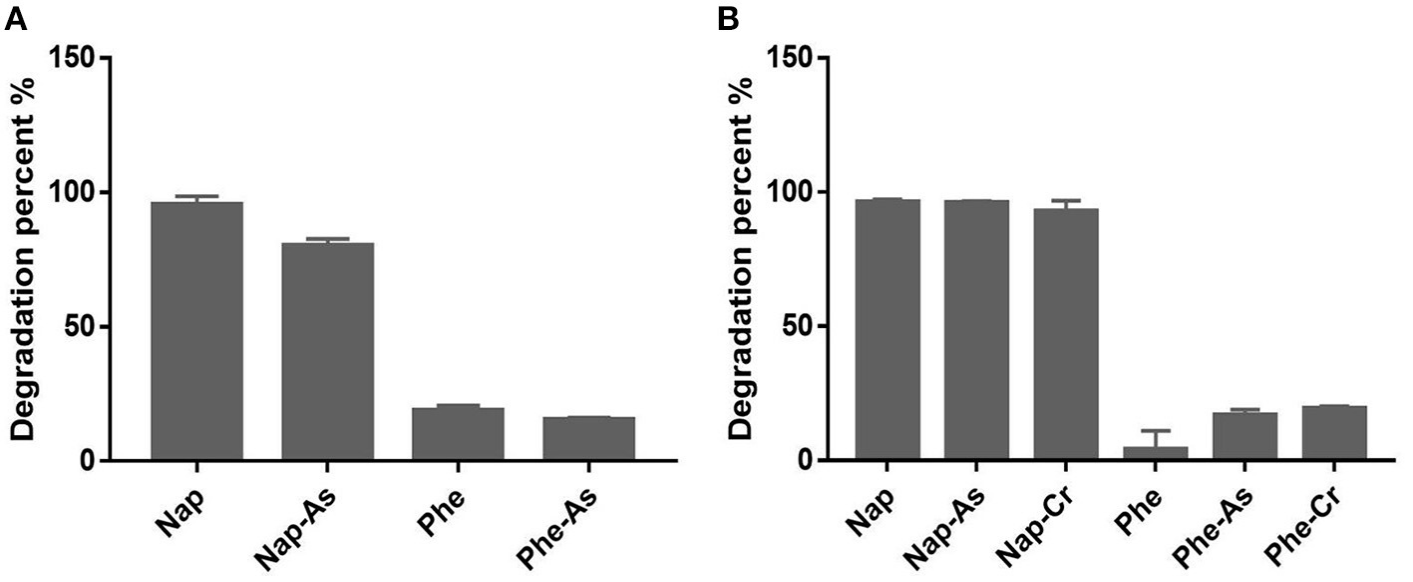
Degradation of naphthalene and phenanthrene by H39-1-10^T^ **(A)** and H39-3-25^T^ **(B)** in the presence or absence of heavy metals. Nap, naphthalene; Phe, phenanthrene; Nap-As and Phe-As mean the medium for PAH degradation was added 0.5 mM sodium arsenite; Nap-Cr and Phe-Cr indicate that the medium for PAH degradation was added 5 mg/L potassium dichromate.

Although originated from polluted soil samples, strains H3Y2-19a^T^, H3SJ31-1^T^, and H3M29-4^T^ could not use any of the tested aromatic compounds. Strains H39-1-10^T^ and H39-3-25^T^ showed observable growth as the sole carbon source using naphthalene, 2,5-dihydroxybenzoic, *p*-hydroxybenzoic, protocatechuic, salicylic, phthalic, and benzoic acids, and they were further tested for degradation of naphthalene in the presence of arsenite or dichromate (Figure 3). The results showed that H39-1-10^T^ could catabolize 80.2% of naphthalene in the presence of 0.5mM arsenite, and H39-3-25^T^ eliminated more than 92% of naphthalene, regardless of adding heavy metals or not. According to the genome annotation (Supplementary Table S4), H39-1-10^T^ and H39-3-25^T^ were annotated naphthalene 1,2-dioxygenase (Selifonov et al., 1996), which is the critical enzyme for the first catalytic reaction step of degrading PAHs. The genotypes of H39-1-10^T^ and H39-3-25^T^ agreed with the growth phenotype of utilizing naphthalene as a carbon source. H39-1-10^T^ and H39-3-25^T^ were later identified as species of *Sphingomonas* and *Sphingobium* that are well known for pollutant degraders (Ghosal et al., 2016).

### 3.5 Comparative genome and genomic synteny analyses

**Figure 4:**
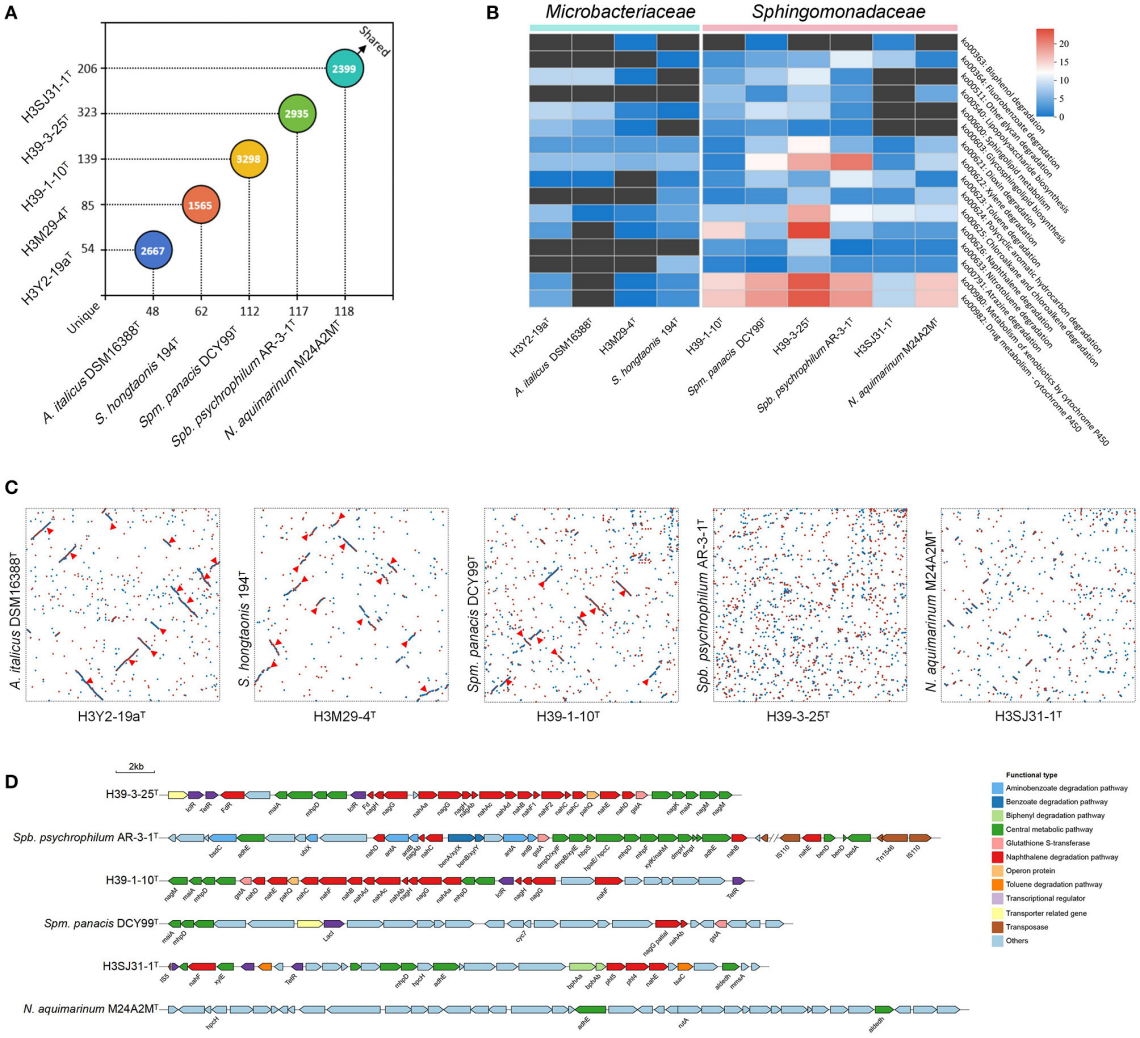
Analysis of orthologous genes and genomic synteny between the five strains and their nearest phylogenomic type strain neighbors. **(A)** The distribution of shared and unique orthologous gene clusters between the five queried strains (y-axis) and their respective reference strains (x-axis). The numbers on the axis indicate the unique orthologous clusters of each strain. The numbers in the circles indicate the shared orthologous clusters between two comparative strains. **(B)** The richness of functional genes related to the biodegradation and metabolism of xenobiotics based on the KEGG annotation in pathway level 3. The richness is illustrated by heatmap analysis. The color indicates the number of annotated genes. The pathway KO number and their functional descriptions are shown on the right side of the heatmap diagram. **(C)** The genomic synteny between the five queried strains (x-axis) and their respective reference strains (y-axis). The syntenic regions are displayed by the dot plot analysis using MUMmer software in a Promer way. Red dots represent forward synteny. Blue dots represent reverse synteny. The syntenic blocks are signed with a red triangle. **(D)** The functional gene clusters for metabolizing xenobiotics in the strains H39-1-10^T^, H39-3-25^T^, H3SJ31-1^T^, and their respective reference strains. The genes that perform roles in one pathway are shown with the same color. The arrows point in the direction of gene expression.

The comparative genomic analyses were carried out to reveal the genomic synteny and diversity between the five strains and their relatives. The nearest phylogenomic type strain neighbors, namely, *Agromyces italicus* DSM 16388^T^ to H3Y2-19a^T^, *Salinibacterium hongtaonis* 194^T^ to H3M29-4^T^, *Sphingomonas panacis* DCY99^T^ to H39-1-10^T^, *Sphingobium psychrophilum* AR-3-1^T^ to H39-3-25^T^, and *Novosphingobium aquimarinum* M24A2M^T^ to H3SJ31-1^T^, which were isolated from catacombs, feces of Tibetan antelopes, rhizosphere, Arctic soil, and seawater samples, respectively, were chosen for comparative genomic analysis. Through comparative analysis of the orthologous relationship of predicted protein-coding genes, we found remarkable gene overlaps among these investigated strains' genomes (Figure 4A). At the level of protein sequence, analysis using OrthoVenn2 revealed that there are 2667, 1565, 3298, 2935, and 2399 orthologous clusters shared by H3Y2-19a^T^, H3M29-4^T^, H39-1-10^T^, H39-3-25^T^, and H3SJ31-1^T^ compared to their relatives, respectively. All the five strains in this study possess more unique orthologous clusters than their relatives. Among the unique clusters of the five studied strains, the annotated known genes account for 46.2%–75.0% (data not shown). Thus, there are still many unknown protein-coding genes in their genomes that need to be explored. From the dot plot of genomic synteny analysis based on protein sequences, we observed the syntenic regions of DNA sequence shared by the studied strains and their respective relatives (Figure 4C). The overall results showed that H3Y2-19a^T^, H3M29-4^T^, and H39-1-10^T^ shared more syntenic blocks (12, 13, and 9 for each) with their respective relatives than H39-3-25^T^ and H3SJ31-1^T^. Although H39-3-25^T^ has a much more analogous sequence with *Spm. panacis* DCY99^T^, no remarkable syntenic blocks were observed. The observations are also supported by the synteny analysis using Mauve software based on DNA sequences, as shown in Supplementary Figure S2. The results suggest that the level of genomic synteny for H3Y2-19a^T^, H3M29-4^T^, and H39-1-10^T^ with their respective relatives is much higher than that for H39-3-25^T^ and H3SJ31-1^T^. The fact that the majority of the genome regions of H39-3-25^T^ and H3SJ31-1^T^ were not in syntenic blocks suggests that dramatic gene rearrangements occurred in their genomes after they diverged from their most recent common ancestor. On the other hand, there are still extensive syntenic DNA sequence orders existing between them and their relatives. These syntenic and dislocated genes may be caused by selective aggregation under evolutionary pressures (Wan, 2019).

As the five strains were isolated from polluted soils, we further comparatively investigated the distribution of functional genes related to the degradation of xenobiotic pollutants between them and their relatives based on the KEGG annotation. The richness of annotated functional genes is shown in Figure 4B. The overall results showed that the bacterial species from the family *Sphingomonadaceae* represented by H39-1-10^T^, H39-3-25^T^, and H3SJ31-1^T^ possess much more functional protein-coding genes than the species from the family *Microbacteriaceae* represented by H3Y2-19a^T^ and H3M29-4^T^, such as genes in the pathways of ko00364, ko00623, ko00624, ko00625, ko00626, and ko00633 for the degradation of fluorobenzoate, toluene, PAHs, chloroalkane and chloroalkene, naphthalene, and nitrotoluene, respectively. These sphingomonads also have numerous genes encoding cytochrome P450 in the pathways of ko00980 and ko00982 to deal with other xenobiotics and drugs. The previous studies showed that strains of sphingomonads usually showed great contaminant-degrading efficiency (Waigi et al., 2015), and thus, they had been widely considered as excellent decomposers of pollutants (Ghosal et al., 2016). The results of this study agree with this opinion at the molecular level. Among the three sphingomonads, the studied species of the genera *Sphingomonas* and *Sphingobium* have more genes than *Novosphingobium* in the pathways of ko00540, ko00603, and ko00600 for lipopolysaccharide and glycosphingolipid biosynthesis and sphingolipid metabolism, which were probably responsible for producing surfactants such as rhamnolipid (Ma et al., 2018; Posada-Baquero et al., 2019), to accelerate the degradation of xenobiotics. The strain H39-1-10^T^ has 15 genes in the ko00626 pathway responsible for naphthalene degradation. The strain H39-3-25^T^ has the most functional genes in the pathways of ko00621 (13), ko00625 (18), ko00626 (24), ko00980 (23), and ko00982 (23) than other strains to degrade dioxin, chloroalkane and chloroalkene, naphthalene, other xenobiotics, and drugs, respectively. It also has 18 functional genes for xylene degradation. Moreover, to investigate the particular mechanism of pollutant degradation of these sphingomonads, specifically the pathway of naphthalene degradation, the functionally related gene clusters were drawn in Figure 4D. As can be observed, both the strains H39-3-25^T^ and H39-1-10^T^ have intact upstream metabolic pathway with the required genes *nah*A, *nah*B, *nah*C, *nah*D, *nah*E, and *nah*F progressively transforming naphthalene to salicylate. The salicylate was then transformed to gentisate using *nag*G and *nag*H and afterward entered the tyrosine metabolism pathway to be mineralized or assimilated. There are also some genes of the central metabolic pathway of PAHs existing around the gene cluster such as *mai*A, *nag*M, *nag*K, and *mhp*D. In contrast, their closest relatives *Spm. panacis* DCY99^T^ and *Spb. psychrophilum* AR-3-1^T^ all lack the intact metabolic pathway of naphthalene. Although *Spb. psychrophilum* AR-3-1^T^ have some scattered genes such as *nah*B, *nah*C, *nah*D, and *nah*E, it is lack of the most critical gene *nah*A that encodes the naphthalene 1,2-dioxygenase for the first oxidation step of naphthalene degradation (Selifonov et al., 1996). Strain H3SJ31-1^T^ only possesses *nah*E and *nah*F in the cluster that could not solely complete the degradation process of naphthalene, and no upstream metabolic pathway gene clusters were found in its closest relative *N. aquimarinum* M24A2M^T^. The interpretations of the comparative genome analysis are in agreement with the results of the phenotype test of naphthalene degradation, as described above. Compared with their respective closest relatives which were isolated from non-polluted samples, H39-1-10^T^ and H39-3-25^T^ evolve much more genes to construct the intact pathway of naphthalene degradation, suggesting that the genes for pollutants degradation were enriched in the heavy PAH-polluted soil environments.

### 3.6 Five novel bacterial taxa and their species descriptions

**Figure 5:**
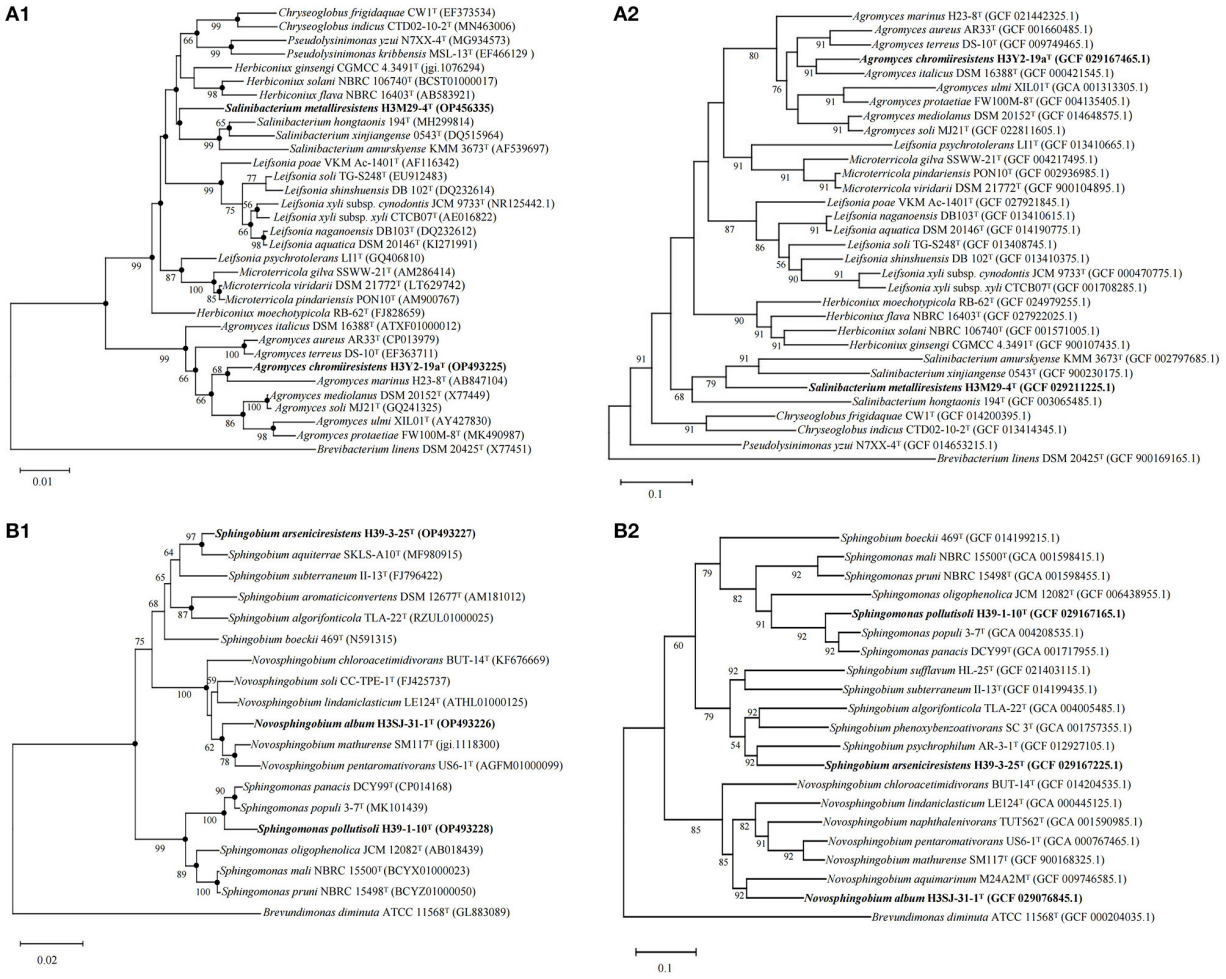
Phylogenetic and phylogenomic trees of the five bacteria constructed on the basis of the 16S rRNA gene sequences and whole genomes using the neighbor-joining algorithm showing the relationships of the five novel taxa to their closely related type bacterial strains. **(A)** The phylogenetic **(A1)** and phylogenomic **(A2)** trees of strains H3Y2-19a^T^, H3M29-4^T^, and their closely related species in the family *Microbacteriaceae*, and the sequence of *Brevibacterium linens* DSM 20425^T^ (X77451) was used as an out-group; **(B)** the phylogenetic **(B1)** and phylogenomic **(B2)** trees of strains H39-1-10^T^, H39-3-25^T^, H3SJ-31-1^T^, and their closely related species in the family *Sphingomonadaceae*, and the sequence of *Brevundimonas diminuta* ATCC 11568^T^ (GL883089) was used as an out-group. GenBank accession numbers are given in parentheses. Bootstrap percentages (>50%) based on 1,000 replicates are shown at the nodes. Phylogenetic trees based on the maximum-likelihood and the maximum-parsimony methods with 1,000 bootstraps were also reconstructed (see Supplementary Figure S3). The filled circles indicate the nodes supported by all three methods regardless of bootstrap percentages. Bar, 0.01 substitutions per nucleotide position for **(A1)**; bar, 0.02 substitutions per nucleotide position for **(B1)**; bar, 0.1 substitutions per nucleotide position for **(A2, B2)**.

**Figure 6:**
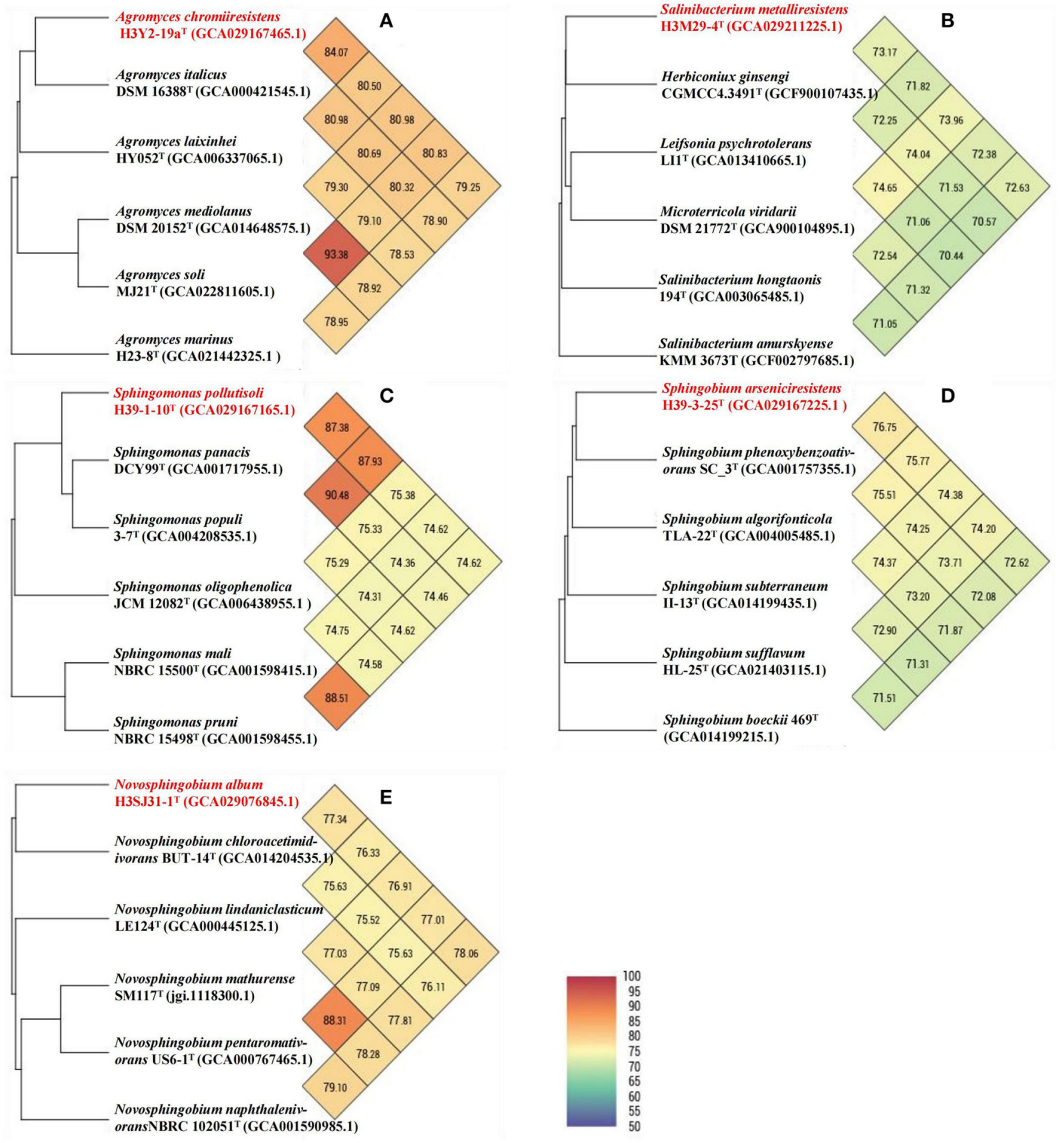
UPGMA phylogenetic trees and ANI heat maps based on whole genomes. Each of the UPGMA phylogenetic trees and the ANI heat maps displays the connections between a novel bacterial taxon and their respective close relatives: **(A)** H3Y2-19a^T^; **(B)** H3M29-4^T^; **(C)** H39-1-10^T^; **(D)** H39-3-25^T^; and **(E)** H3SJ31-1^T^. The novel taxon names proposed in this study are shown in red. GenBank accession numbers of the genomes are shown in parentheses.

Based on the 16S rRNA gene and genome sequences analysis, we further studied the phylogenetic relationships of each bacterial isolate with their closely related and validly nominated bacterial taxa, and the phylogenetic trees are shown in Figure 5. The UPGMA dendrogram trees based on the ANI scores of their genomes were generated and are shown in Figure 6. The dDDH values were calculated and are shown in Supplementary Tables S2, S3. According to the chemotaxonomic characteristics and the results from DNA molecule analysis, we proposed that H3Y2-19a^T^, H3M29-4^T^, H39-1-10^T^, H39-3-25^T^, and H3SJ31-1^T^ represent novel species.

#### 3.6.1 Strain H3Y2-19a^*T*^

The phylogenetic trees revealed that H3Y2-19a^T^ clustered members of *Agromyces* genus (Figures 5A1, A2). The close relatives to H3Y2-19a^T^ were *A. italicus* DSM 16388^T^ (97.58%, 16S rRNA gene identity), *A. marinus* H23-8^T^ (97.22%), *A. mediolanus* DSM 20152^T^ (97.72%), and *A. soli* MJ21^T^ (97.61%). The ANI and dDDH values between H3Y2-19a^T^ and *A. italicus* DSM 16388^T^ were 84.07% and 27.1 %, respectively (Table 1 and Figure 6A), which were all below the threshold for differentiating two species (ANI, 95% and dDDH, 70%; Goris et al., 2007; Richter and Rosselló-Móra, 2009). The major cellular fatty acids of H3Y2-19a^T^ were iso-C_15:0_ (13.20%), iso-C_16:0_ (20.77%), anteiso-C_15:0_ (35.17%), and anteiso-C_17:0_ (23.34%) (Supplementary Table S2), which were consistent with the description of the genus *Agromyces*. H3Y2-19a^T^ contained polar lipids of diphosphatidylglycerol, phosphatidylglycerol, and unknown glycolipids (Supplementary Figure S1). The DNA G+C content was 71.22 mol%, which is in the range (>70 mol%; Jurado et al., 2005) of the *Agromyces* genus (Supplementary Table S2). Based on the results of phylogenetic, phylogenomic, and phenotypic characterizations, we concluded that strain H3Y2-19a^T^ represents a novel species affiliated to the genus *Agromyces*, and the name *Agromyces chromiiresistens* sp. nov. is proposed.

##### 3.6.1.1 Description of *Agromyces chromiiresistens* sp. nov

*Agromyces chromiiresistens* (chro.mi.i.re.sis.tens. N.L. neut. n. *chromium*, chromium (Cr); L. pres. part. *resistens*, resisting; N.L. part. adj. *chromiiresistens*, chromium resisting, denoting the type strain is chromium-resistant).

Cells are Gram-positive, aerobic, non-endospore-forming, and rods with a size of approximately 1.26–2.58 × 0.39–0.57 μm. Colonies grown on R2A agar plates are circular, convex, and white after cultivating for 2 days, and the color changes to fluorescent yellow as the cultivation prolongs. The cells metabolize dextrin, _D_-maltose, _D_-cellobiose, sucrose, stachyose, _D_-raffinose, N-acetyl-_D_-glucosamine, N-acetyl-β-_D_-mannosamine, α-_D_-glucose, _D_-mannose, N-acetyl-_D_-galactosamine, _D_-fructose, _D_-galactose, _L_-rhamnose, myo-inositol, glycerol, _L_-histidine, pectin, _D_-gluconic acid, p-hydroxy-phenylacetic acid, _L_-lactic acid, Tween 40, β-hydroxy-_D_, _L_-butyric acid, _D_-turanose, _D_-salicin, _L_-fucose, inosine, glycyl-_L_-proline, and _L_-glutamic acid. They grow at pH 6.0 but fail to grow when pH is lower than pH 5.0. The cells tolerate 1% NaCl but not 4–8% NaCl and tolerate 1% sodium lactate, rifamycin SV, lincomycin, vancomycin, nalidixic acid, lithium chloride, potassium tellurite, aztreonam, and sodium butyrate. They resist 0.5–10.0 mM sodium arsenite and 5–100 mg/L potassium. The major polar lipids are diphosphatidylglycerol, phosphatidylglycerol, unknown glycolipids, and unknown lipid. The genome size is 4.09 Mbp, and the DNA G+C content is 71.22 mol%.

The type strain is H3Y2-19a^T^ (=CGMCC 1.61332^T^), isolated from a polluted soil sample. The GenBank accession numbers for the 16S rRNA gene sequence and genome sequence of the type strain are OP493225 and JARFNL000000000, respectively.

#### 3.6.2 Strain H3M29-4^*T*^

The phylogenetic trees revealed that H3M29-4^T^ clustered members of *Salinibacterium* genus (Figures 5A1, A2). The close relatives to H3M29-4^T^ were *S. hongtaonis* 194^T^ (97.51 %, 16S rRNA gene identity), *S. amurskyense* KMM 3673^T^ (96.94%), and *S. xinjiangense* 0543^T^ (96.61%). The ANI and dDDH values between H3M29-4^T^ and *S. hongtaonis* 194^T^ were 72.38 and 14.0 %, respectively (Table 1 and Figure 6B), which were all below the threshold for differentiating two species. At the date of this description, three species of the *Salinibacterium* genus have been described. The major cellular fatty acids of H3M29-4^T^ were anteiso-C_15:0_ (41.05%), iso-C_16:0_ (25.60%), and anteiso-C_17:0_ (27.46%) (Supplementary Table S2), which were consistent with the description of the genus *Salinibacterium*. H3M29-4^T^ contained polar lipids of diphosphatidylglycerol, phosphatidylglycerol, unknown glycolipids, and unknown lipid (Supplementary Figure S1). The DNA G+C content was 69.07 mol%, which was consistent with other members of the *Salinibacterium* genus (Supplementary Table S2). Based on the results of phylogenetic, phylogenomic, and phenotypic characterizations, we concluded that strain H3M29-4^T^ represents a novel species affiliated to the genus *Salinibacterium*, and thus the name *Salinibacterium metalliresistens* sp. nov. is proposed.

##### 3.6.2.1 Description of *Salinibacterium metalliresistens* sp. nov

*Salinibacterium metalliresistens* (me.tal.li.re.sis'tens. L. neut. n. *metallum*, metal; L. pres. part. *resistens*, resisting; N.L. part. adj. *metalliresistens*, metal-resistant, referring to the ability of the bacterium to resist heavy metals).

Cells are Gram-positive, aerobic, and rods with a size of approximately 1.73–2.86 × 0.30–0.42 μm. Colonies grown on R2A agar plates are smooth, translucent, pale yellow, and circular after cultivating 3–4 days. The cells metabolize dextrin, _D_-maltose, _D_-trehalose, _D_-cellobiose, sucrose, _D_-turanose, α-_D_-lactose, _D_-salicin, α-_D_-glucose, _D_-mannose, _D_-fructose, _D_-galactose, _L_-fucose, inosine, pectin, glucuronamide, _L_-lactic acid, Tween 40, acetoacetic acid, and acetic acid. They grow at pH 6.0 but fail to grow when pH is lower than pH 5.0. The cells tolerate 1% NaCl but not 4-8% NaCl and tolerate 1% sodium lactate, nalidixic acid, and potassium tellurite. They resist 0.5 mM sodium arsenite and 5-20 mg/L potassium dichromate. The major cellular fatty acids are anteiso-C_15:0_, iso-C_16:0_, and anteiso-C_17:0_. The major polar lipids are diphosphatidylglycerol, phosphatidylglycerol, unknown glycolipids, and unknown lipid. The genome size is 3.07 Mbp, and the DNA G+C content is 69.07 mol%.

The type strain is H3M29-4^T^ (=CGMCC 1.61335^T^), isolated from a polluted soil sample. The GenBank accession numbers for the 16S rRNA gene sequence and genome sequence of the type strain are OP456335 and JARGEM000000000, respectively.

#### 3.6.3 Strain H39-1-10^*T*^

The phylogenetic trees revealed that H39-1-10^T^ clustered members of *Sphingomonas* genus (Figures 5B1, B2). The close relatives to H39-1-10^T^ were *Spm. panacis* DCY99^T^ (98.36%, 16S rRNA gene identity), *Spm. populi* 3-7^T^ (97.61%), *Spm. oligophenolica* JCM 12082^T^ (96.30%), and *Spm. mali* NBRC 15500^T^ (96.36%). The ANI and dDDH values between H39-1-10^T^ and *Spm. panacis* DCY99^T^ were 87.38% and 34.2%, respectively (Table 2 and Figure 6C), which were all below the threshold for differentiating two species. At the date of this description, 152 species of the *Sphingomonas* genus have been described. The major cellular fatty acids of H39-1-10^T^ were summed feature 8 (C_18:1_ ω7c/C_18:1_ ω6c, 51.19%), C_14:0_ 2-OH (30.14%), and C_16:0_ (9.85%) (Supplementary Table S3), which were consistent with the description of the genus *Sphingomonas*. H39-1-10^T^ contained polar lipids of diphosphatidylglycerol, phosphatidylglycerol, phosphatidylethanolamine, phosphatidylcholine, sphingoglycolipid, unknown aminophospholipid, unknown lipid, and unknown glycolipid (Supplementary Figure S1). The DNA G+C content was 66.15 mol%, which is in the range (62–68 mol%; Ohta et al., 2004) of the *Sphingomonas* genus (Supplementary Table S3). Based on the results of phylogenetic, phylogenomic, and phenotypic characterizations, we concluded that strain H39-1-10^T^ represents a novel species affiliated to the genus *Sphingomonas* and the name *Sphingomonas pollutisoli* sp. nov. is proposed.

##### 3.6.3.1 Description of *Sphingomonas pollutisoli* sp. nov

*Sphingomonas pollutisoli (pol.lu.ti.so'li*. L. masc. perf. part. *pollutus*, contaminated, soiled; L. neut. adj. *solum*, soil; N.L. gen. neut. n. *pollutisoli*, of polluted soil).

Cells are Gram-negative, aerobic, and rods with a size of approximately 2.14–3.33 × 0.95–1.18 μm. Colonies grown on R2A agar plates are circular, milk-white, and non-transparent. The cells metabolize dextrin, _D_-maltose, _D_-trehalose, _D_-cellobiose, α-_D_-lactose, α-_D_-glucose, _D_-mannose, N-acetyl-_D_-galactosamine, _D_-fructose, _D_-galactose, _D_-fucose, _L_-fucose, _L_-rhamnose, glycerol, glycyl-_L_-proline, _L_-aspartic acid, _L_-glutamic acid, _D_-galacturonic acid, _L_-galactonic acid lactone, _D_-gluconic acid, _D_-glucuronic acid, glucuronamide, _L_-lactic acid, Tween 40, β-hydroxy-_D, L_-butyric acid, α-keto-butyric acid, 2,5-dihydroxybenzoic acid, *p*-hydroxybenzoic acid, protocatechuic acid, salicylic acid, phthalic acid, and benzoic acid. They grow at pH 6.0 but fail to grow when pH is lower than pH 5.0. The cells do not tolerate 1–8% NaCl but resist 0.5 mM sodium arsenite. They degrade naphthalene even in the presence of sodium arsenite. The major cellular fatty acids are sphingoglycolipid, diphosphatidylglycerol, phosphatidylethanolamine, phosphatidylglycerol, phosphatidylcholine, unknown glycolipids, unknown aminophospholipid, and unknown lipids. The major fatty acids are C_16:0_, summed feature 8 (C_18:1_ ω7c/C_18:1_ ω6c), and C_14:0_ 2-OH. The genome size is 5.55 Mbp, and DNA G+C content is 66.15 mol%.

The type strain is H39-1-10^T^ (=CGMCC 1.61325^T^), isolated from a polluted soil sample. The GenBank accession numbers for the 16S rRNA gene sequence and genome sequence of the type strain are OP493228 and JARFNJ000000000, respectively.

#### 3.6.4 Strain H39-3-25^*T*^

The phylogenetic trees revealed that H39-3-25^T^ clustered members of *Sphingobium* genus (Figures 5B1, B2). The close relatives to H39-3-25^T^ were *Spb. aquiterrae* SKLS-A10^T^ (97.95%, 16S rRNA gene identity), *Spb. aromaticiconvertens* DSM 12677^T^ (97.15%), *Spb. algorifonticola* TLA-22^T^ (96.86%), and *Spb. subterraneum* II-13^T^ (96.64%). The ANI and dDDH values between H39-3-25^T^ and *Spb. algorifonticola* TLA-22^T^ were 74.38 and 20.8%, respectively (Table 2 and Figure 6D), which were all below the threshold for differentiating two species. At the date of this description, 44 species of the *Sphingobium* genus have been described. The major cellular fatty acids of H39-3-25^T^ were summed feature 8 (C_18:1_ ω7c/C_18:1_ ω6c, 50.50%), C_14:0_ 2-OH (15.94%), and C_16:0_ (10.80%) (Supplementary Table S3), which were consistent with the description of the genus *Sphingobium*. H39-3-25^T^ contained polar lipids of diphosphatidylglycerol, phosphatidylethanolamine, phosphatidylglycerol, phosphatidyl monomethylethanolamine, sphingoglycolipid, and unknown phospholipids (Supplementary Figure S1). The DNA G+C content was 63.57 mol%, which is in the range (62–67 mol%; Lee et al., 2015) of the *Sphingobium* genus (Supplementary Table S3). Based on the results of phylogenetic, phylogenomic, and phenotypic characterizations, we concluded that strain H39-3-25^T^ represents a novel species affiliated to the genus *Sphingobium* and the name *Sphingobium arseniciresistens* sp. nov. is proposed.

##### 3.6.4.1 Description of *Sphingobium arseniciresistens* sp. nov

*Sphingobium arseniciresistens* (ar.se.ni.ci.re.sis'tens. N.L. neut. adj. *arsenicum*, arsenic; L. pres. part. *resistens*, resisting; N.L. part. adj. *arseniciresistens*, arsenic resisting, referring to the arsenic resistance of the bacterium).

Cells are Gram-negative, aerobic, motile by means of polar flagellum, and rods with a size of approximately 1.47–2.62 × 0.68–0.81 μm. Colonies grown on R2A agar plates are pale yellow, viscous, and non-transparent. The cells metabolize dextrin, _D_-maltose, _D_-cellobiose, gentiobiose, α-_D_-lactose, _D_-melibiose, β-methyl-_D_-glucoside, _D_-salicin, N-acetyl-_D_-glucosamine, α-_D_-glucose, _D_-mannose, _D_-fructose, _D_-galactose, _D_-fucose, _L_-rhamnose, gelatin, glycyl-_L_-proline, _L_-glutamic acid, _D_-galacturonic acid, _L_-galactonic acid lactone, _D_-glucuronic acid, quinic acid, _L_-malic acid, Tween 40, acetoacetic acid, acetic acid, _D_-trehalose, sucrose, _D_-turanose, N-acetyl-β-_D_-mannosamine, 3-methyl glucose, _L_-fucose, _D_-glucose-6-PO_4_, _D_-fructose-6-PO_4_, _D_-aspartic acid, _L_-aspartic acid, _L_-histidine, _L_-pyroglutamic acid, pectin, glucuronamide, methyl pyruvate, _L_-lactic acid, bromo-succinic acid, β-hydroxy-_D, L_-butyric acid, 2,5-dihydroxybenzoic acid, *p*-hydroxybenzoic acid, protocatechuic acid, salicylic acid, phthalic acid, and benzoic acid. They grow at pH 6.0 but fail to grow when pH is lower than pH 5.0. The cells tolerate 1% NaCl but not 4–8% NaCl and tolerate 1% sodium lactate, _D_-serine, rifamycin SV, lincomycin, tetrazolium blue, and nalidixic acid. They resist 0.5–2.0 mM sodium arsenite and 5–20 mg/L potassium dichromate. The cells degrade naphthalene even in the presence of sodium arsenite or potassium dichromate. The major polar lipids are sphingoglycolipid, diphosphatidylglycerol, phosphatidylethanolamine, phosphatidylglycerol, phosphatidyl monomethylethanolamine, and unknown phospholipids. The major cellular fatty acids include summed feature 8 (C_18:1_ ω7c/C_18:1_ ω6c), C_14:0_ 2-OH, and C_16:0_. The genome size is 6.60 Mbp, and the DNA G+C content is 63.57 mol%.

The type strain is H39-3-25^T^ (=CGMCC 1.61326^T^), isolated from a polluted soil sample. The GenBank accession numbers for the 16S rRNA gene sequence and genome sequence of the type strain are OP493227 and JARFNK000000000, respectively.

#### 3.6.5 Strain H3SJ31-1^*T*^

The phylogenetic trees revealed that H3SJ31-1^T^ clustered members of *Novosphingobium* genus (Figures 5B1, B2). The close relatives to H3SJ31-1^T^ were *N. mathurense* SM117^T^ (97.53%, 16S rRNA gene identity) and *N. soli* CC-TPE-1^T^ (97.60%). The ANI and dDDH values between H3SJ31-1^T^ and *N. mathurense* SM117^T^ were 76.91 and 20.7%, respectively (Table 2 and Figure 6E), which were all below the threshold for differentiating two species. At the date of this description, 58 species of the *Novosphingobium* genus have been described. The major cellular fatty acids of H3SJ31-1^T^ were summed feature 8 (C_18:1_ ω7c/C_18:1_ ω6c, 30.74%) and C_16:0_ (47.67%), and the component of C_16:0_ distinguished this organism from other members of the *Novosphingobium* genus as shown in Supplementary Table S3. H3SJ31-1^T^ contained polar lipids of sphingoglycolipid, diphosphatidylglycerol, phosphatidylethanolamine, phosphatidyl dimethylethanolamine, phosphatidylglycerol, phosphatidylcholine, phosphatidyl monomethylethanolamine, and unknown lipid (Supplementary Figure S1). The DNA G+C content was 66.16 mol%, which is in the range (62–67 mol%; Takeuchi et al., 2001) of the *Novosphingobium* genus (Supplementary Table S3). Based on the results of phylogenetic, phylogenomic, and phenotypic characterizations, we concluded that strain H3SJ31-1^T^ represents a novel species affiliated to the genus *Novosphingobium* and the name *Novosphingobium album* sp. nov. is proposed.

##### 3.6.5.1 Description of *Novosphingobium album* sp. nov

*Novosphingobium album* (al'bum. L. neut. adj. *album* white).

Cells are Gram-positive, aerobic, motile by means of polar flagellum, and ovoid-shaped with a size of approximately 0.89–1.15 × 0.73–0.90 μm. Colonies grown on R2A agar plates are circular, non-transparent, and milk-white after cultivating for 2 days. The cells metabolize glycyl-_L_-proline, _L_-glutamic acid, acetic acid, _L_-aspartic acid, glucuronamide, Tween 40, β-hydroxy-_D, L_-butyric acid, and acetoacetic acid. They grow at pH 6.0 but fail to grow when pH is lower than pH 5.0. The cells do not tolerate 1–8% NaCl. The cells tolerate lincomycin, nalidixic acid, and potassium tellurite. The major polar lipids are sphingoglycolipid, diphosphatidylglycerol, phosphatidylethanolamine, phosphatidylglycerol, phosphatidyl monomethylethanolamine, phosphatidyl dimethylethanolamine, phosphatidylcholine, and unknown lipids. The major cellular fatty acids are C_16:0_ and summed feature 8 (C_18:1_ ω7c/C_18:1_ ω6c). The genome size is 5.03 Mbp, and the DNA G+C content is 66.16 mol%.

The type strain is H3SJ31-1^T^ (=CGMCC 1.61329^T^) isolated from a polluted soil sample. The GenBank accession numbers for the 16S rRNA gene sequence and genome sequence of the type strain are OP493226 and JARESE000000000, respectively.

## Data availability statement

The datasets presented in this study can be found in online repositories. The names of the repository/repositories and accession number(s) can be found in the article/Supplementary material.

## Author contributions

Z-SL: Writing—original draft, Formal analysis, Investigation, Project administration, Software. K-HW: Data curation, Formal analysis, Investigation, software. MC: Data curation. M-LY: Data curation. X-KW: Data curation. H-LM: Data curation. Y-HY: Data curation. L-HW: Data curation. D-FL Conceptualization, Funding acquisition, Resources, Supervision, Writing—review & editing. S-JL: Conceptualization, Funding acquisition, Resources, Supervision, Writing—review & editing.
