# Supplementary material for: Agromyces chromiiresistens sp. nov., Novosphingobium album sp. nov., Sphingobium arseniciresistens sp. nov., Sphingomonas pollutisoli sp. nov., and Salinibacterium metalliresistens sp. nov.: five new members of Microbacteriaceae and Sphingomonadaceae from polluted soil
Source: Front Microbiol. 2023 Nov 28;14:1289110. doi: 10.3389/fmicb.2023.1289110 (PMC10713820; doi:10.3389/fmicb.2023.1289110)
Supplement: Supplementary file 1 [file Data_Sheet_1.pdf]

Supplementary materials for:

***Agromyces chromiiresistens* sp. nov., *Novosphingobium album* sp. nov., *Sphingobium arseniciresistens* sp. nov., and *Sphingomonas pollutisoli* sp. nov., *Salinibacterium metalliresistens* sp. nov., 5 new members of *Microbacteriaceae* and *Sphingomonadaceae* from polluted soil**

Ze-Shen Liu[1†], Ke-Huan Wang[1,3†], Man Cai[1], Mei-Ling Yang[1,3], Xiao-Kang Wang[1,3], Hong-Lin Ma[1,3], Yi-Han Yuan[4], Lin-Huan Wu[1], De-Feng Li*[1,3], Shuang-Jiang Liu*[1,2,3]

1. State Key Laboratory of Microbial Resources and Environmental Microbiology Research Center at Institute of Microbiology, Chinese Academy of Sciences, Beijing 100101, China
2. State Key Laboratory of Microbial Biotechnology, Shandong University, Qingdao 266237, China
3. University of Chinese Academy of Sciences, Beijing 100049, China.
4. School of Life Science, Hebei University, Baoding, Baoding 071002, China.

*Corresponding authors:
Shuang-Jiang Liu (liusj@im.ac.cn) & De-Feng Li (lidefeng@im.ac.cn)
Postal address: Institute of Microbiology, Chinese Academy of Sciences, Beichen Xilu No. 1, Chaoyang District, Beijing 100101, China
Telephone number: +86-010-64807423

Table S1. The quantity of annotated genes related to antibiotics resistance, and metabolism of carbohydrates and amino acids in the 5 novel species.

The genomes were annotated using RAST engine. The quantity of annotated functional genes was list in the table. -, no related genes were annotated.

Strains	H3Y2-19a^T	H3M29-4^T	H39-1-10^T	H39-3-25^T	H3SJ31-1^T
**Resistance to antibiotics**					
Anti fluoroquinolones	2	2	2	3	2
Beta-lactamase	-	-	1	3	1
Multidrug resistance efflux pumps	2	-	9	-	-
**Carbohydrates metabolism**					
Central carbohydrate metabolism	97	100	110	107	115
Monosaccharides	52	17	55	35	4
Di- and oligosaccharides	19	16	21	8	3
Aminosugars	18	6	8	8	-
**Organic acids**	14	14	12	9	11
**Amino acids and derivatives**	325	235	265	378	314

Table S2. Cellular fatty acid compositions and genomic features of 2 novel species of *Microbacteriaceae*, and differentiation to their closely related species. Strains: 1, H3Y2-19a^T; 2, *Agromyces mediolanus* DSM 20152^T (Suzuki et al., 1996); 3, *Agromyces soli* MJ21^T (Lee et al., 2011); 4, *Agromyces italicus* DSM 16388^T (Jurado et al., 2005); 5, *Agromyces marinus* H23-8^T (Hamada et al., 2014); 6, H3M29-4^T; 7, *Salinibacterium xinjiangense* 0543^T (Zhang et al., 2008); 8, *Salinibacterium amurskyense* KMM 3673^T (Han et al., 2003); 9, *Salinibacterium hongtaonis* 194^T (Lu et al., 2023). Data in columns 2–5 and 7-9 were obtained from Suzuki K *et al*. (1996), Lee M *et al*. (2011), Jurado V *et al*. (2005), Hamada M *et al*. (2014), Zhang D-C *et al*. (2008), Han SK *et al*. (2003) and Lu C-Y *et al*. (2023), respectively. /, not reported; TR, trace amount; ND, not detected.

Strains	1	2	3	4	5	6	7	8	9
$C_{14:0}$	TR	ND	/	/	/	ND	/	/	/
$C_{16:0}$	1.30	2	1.1	1.7	TR	1.38	TR	/	1.6
$C_{17:0}$	ND	/	/	/	/	TR	/	/	/
$C_{18:0}$	ND	ND	/	/	/	TR	/	/	/
iso-$C_{14:0}$	1.21	/	1.1	/	TR	1.8	10.25	14.7	ND
**iso-$C_{15:0}$**	**13.20**	**2**	**7.4**	**14.2**	**3.7**	TR	TR	6.6	4.0
**iso-$C_{16:0}$**	**20.77**	**20**	**18.1**	**14.7**	**18.2**	**25.6**	**21.13**	**34.7**	**15.0**
iso-C17:0	4.70	ND	1.7	/	1.4	TR	TR	/	/
iso-C18:0	ND	/	/	/	/	TR	/	/	/
iso-15:1 G	TR	/	/	/	/	ND	/	/	/
**anteiso-$C_{15:0}$**	**35.17**	**33**	**46.9**	**50.9**	**41.8**	**41.05**	**58.36**	**40.4**	**45.4**
**anteiso-$C_{17:0}$**	**23.34**	**40**	**23.7**	**15.3**	**33.4**	**27.46**	**7.16**	**2.8**	**28.3**
anteiso-$C_{15:1}$ A	TR	/	/	/	/	ND	1.67	/	/
DNA G+C content (mol%)	71.22	72.30	73.40	70.80	72.50	69.07	63.90	61.90	64.10
Identity of 16S rRNA gene (%)		97.72	97.61	97.58	97.22		96.61	96.94	97.51
ANI (%)		80.98	80.83	84.07	/		75.52	72.63	72.38
dDDH (%)		23.8	23.7	27.1	/		16.7	14.0	14.0

Identity of 16S rRNA gene, ANI and dDDH refer to the value of comparing the 16S rRNA gene sequence or genome sequence with their respective closely related type strains.

Table S3. Cellular fatty acid compositions and genomic features of 3 novel species of *Sphingomonadaceae*, and differentiation to their closely related species. Strains: 1, H39-1-10^{T}; 2, *Sphingomonas panacis* DCY99^{T} (Singh et al., 2015); 3, *Sphingomonas oligophenolica* JCM 12082^{T} (Singh et al., 2015); 4, *Sphingomonas populi* 3-7^{T} (Li et al., 2020); 5, H39-3-25^{T}; 6, *Sphingobium aquiterrae* SKLS-A10^{T} (Revesz et al., 2018); 7, *Sphingobium algorifonticola* TLA-22^{T} (Chen et al., 2020); 8, *Sphingobium subterraneum* II-13^{T} (Lee et al., 2015); 9, H3SJ31-1^{T}; 10, *Novosphingobium soli* CC-TPE-1^{T} (Kampfer et al., 2011); 11, *Novosphingobium mathurense* SM117^{T} (Gupta et al., 2009); 12, *Novosphingobium lindaniclasticum* LE124^{T} (Saxena et al., 2013). Data in columns 2–4, 6-8 and 10-12 were obtained from Singh P *et al.* (2015), Singh P *et al.* (2015), Li Y *et al.* (2020), Revesz F V *et al.* (2018), Chen W-M M *et al.* (2020), Lee J-C *et al.* (2015), Kampfer P *et al.* (2011), Gupta SK *et al.* (2009) and Saxena A *et al.* (2013), respectively. /, not reported; TR, trace amount (< 1.0%); ND, not detected. Major fatty acid components are indicated with bold text.

Strains	1	2	3	4	5	6	7	8	9	10	11	12
$C_{10:0}$	ND	1.9	1.1	TR	ND	/	/	/	ND	/	/	/
$C_{12:0}$	ND	/	/	4.6	ND	/	/	TR	ND	/	/	TR
$C_{14:0}$	ND	1.3	1.3	2.1	TR	/	ND	TR	2.41	ND	1.9	1.0
$C_{15:0}$	ND	/	/	/	ND	/	/	1.9	ND	TR	/	/
**$C_{16:0}$**	**9.85**	**13.0**	**14.0**	**11.0**	**10.80**	**6.3**	**5.1**	**15.5**	**47.67**	**7.3**	**8.4**	**6.7**
$C_{17:0}$	ND	/	/	ND	ND	/	1.8	/	TR	ND	ND	TR
$C_{18:0}$	ND	/	/	1.2	1.14	/	ND	3.2	5.65	TR	1.6	TR
cyclo-$C_{19:0}$ ω8c	ND	/	/	/	TR	/	/	/	1.11	/	/	/
iso-$C_{10:0}$	ND	3.4	2.2	ND	ND	/	/	/	ND	/	/	/
iso-$_{16:0}$ 3-OH	1.04	/	/	/	ND	/	/	/	ND	ND	/	/
iso-$_{17:0}$ 3-OH	ND	/	/	2.3	ND	/	/	/	ND	/	/	/
**$C_{14:0}$ 2-OH**	**30.14**	**11.4**	**10.6**	**4.4**	**15.94**	**12.8**	**5.2**	**16.3**	2.68	8.2	7.5	9.4
$C_{15:0}$ 2-OH	ND	/	/	ND	TR	1.5	6.1	4.6	ND	2.4	ND	ND
$C_{15:0}$ 3-OH	ND	/	/	/	1.57	/	/	/	ND	/	/	/
$C_{16:0}$ 2-OH	ND	/	/	/	TR	/	/	1.3	TR	1.0	3.5	2.1
$C_{18:0}$ 3-OH	ND	/	/	/	ND	/	/	/	TR	/	/	/
$C_{16:1}$ ω5c	TR	/	/	/	1.43	1.5	1.1	TR	TR	1.9	3.8	3.3
$C_{17:1}$ ω6c	1.12	ND	ND	ND	1.57	6.6	26.1	6.7	TR	11.0	1.8	4.9

$C_{17:1}$ ω8c	ND	/	/	ND	TR	/	4.3	/	ND	1.7	ND	TR
$C_{18:1}$ ω5c	1.81	ND	ND	1.1	TR	TR	ND	ND	TR	TR	/	/
$C_{18:1}$ ω9c	ND	/	/	/	ND	/	/	/	1.60	/	/	/
11-methyl-$C_{18:1}$ ω7c	4.13	7.2	10.2	8.1	7.07	1.9	3.3	10.0	2.24	7.6	/	/
$C_{18:3}$ ω6c (6, 9, 12)	ND	3.1	1.6	TR	ND	/	/	/	ND	/	/	/
Summed feature 3	ND	2.1	4.1	1.6	6.87	9.0	7.6	12.4	2.03	6.3	11.5	19.9
**Summed feature 8**	**51.19**	**55.9**	**50.0**	**60.8**	**50.5**	**55.7**	**35.9**	**23.1**	**30.74**	**49.9**	**57.9**	**49.1**
DNA G+C content (mol%)	66.15	64.40	64.20	65.10	63.57	65.90	63.20	63.50	66.16	/	63.30	60.70
Identity of 16S rRNA gene (%)		98.36	96.30	97.61		97.95	96.86	96.64		97.60	97.53	97.38
ANI (%)		87.38	75.40	87.93		/	75.77	74.38		/	76.91	76.33
dDDH (%)		34.2	20.0	35.2		/	20.7	20.8		/	20.7	21

Identity of 16S rRNA gene, ANI and dDDH refer to the value of comparing the 16S rRNA gene sequence or genome sequence with their respective closely related type strains. Summed features represent the integration of two or three fatty acids which cannot be differentiated by the MIDI system. Summed feature 3 included $C_{16:1}$ ω7c and/or $C_{16:1}$ ω6c; summed feature 8 included $C_{18:1}$ ω7c and/or $C_{18:1}$ ω6c.

Table S4. The quantity of annotated genes related to heavy metals resistance and aromatic compounds metabolism in the 5 novel species.

The genomes were annotated using DIAMOND software by referring to databases including KEGG, COG, NR, SwissProt and Pfam. The quantity of annotated functional genes was list in the table. -, no related genes were annotated.

Strains	H3Y2-19a^{T}	H3M29-4^{T}	H39-1-10^{T}	H39-3-25^{T}	H3SJ31-1^{T}
**Enymes for arsenic resistance**					
ArsA	-	-	-	-	-
ArsB	-	-	1	-	-
Other arsenic transporters	1	1	2	1	3
**Enymes for chromium resistance**					
ChrB	-	-	1	-	-
ChrR	-	-	1	-	-
Chromate transporter	-	-	-	1	-
**Enymes for aromatic compounds metabolism**					
Naphthalene 1,2-dioxygenase	-	-	4	11	-
Cytochrome P450	-	-	1	4	-
Peripheral pathways for catabolism of aromatic compounds	2	8	9	26	1
Metabolism of central aromatic intermediates	11	7	16	56	21

References

Chen, W.M., Guo, Y.P., Sheu, C., and Sheu, S.Y. (2020). *Sphingobium algorifonticola* sp. nov., isolated from a cold spring. *Int. J. Syst. Evol. Microbiol.* 70, 309-316. doi: 10.1099/ijsem.0.003755.

Gupta, S.K., Lal, D., and Lal, R. (2009). *Novosphingobium panipatense* sp. nov. and *Novosphingobium mathurense* sp. nov., from oil-contaminated soil. *Int. J. Syst. Evol. Microbiol.* 59, 156-161. doi: 10.1099/ijs.0.65743-0.

Hamada, M., Shibata, C., Tamura, T., and Suzuki, K. (2014). *Agromyces marinus* sp. nov., a novel actinobacterium isolated from sea sediment. *J. Antibiot. (Tokyo)* 67, 703-706. doi: 10.1038/ja.2014.60.

Han, S.K., Nedashkovskaya, O.I., Mikhailov, V.V., Kim, S.B., and Bae, K.S. (2003). *Salinibacterium amurskyense* gen. nov., sp. nov., a novel genus of the family *Microbacteriaceae* from the marine environment. *Int. J. Syst. Evol. Microbiol.* 53, 2061-2066. doi: 10.1099/ijs.0.02627-0.

Jurado, V., Groth, I., Gonzalez, J.M., Laiz, L., Schuetze, B., and Saiz-Jimenez, C. (2005). *Agromyces italicus* sp. nov., *Agromyces humatus* sp. nov. and *Agromyces lapidis* sp. nov.,

isolated from Roman catacombs. *Int. J. Syst. Evol. Microbiol.* 55, 871-875. doi: 10.1099/ijs.0.63414-0.

Kampfer, P., Young, C.C., Busse, H.J., Lin, S.Y., Rekha, P.D., Arun, A.B., et al. (2011). *Novosphingobium soli* sp. nov., isolated from soil. *Int. J. Syst. Evol. Microbiol.* 61, 259-263. doi: 10.1099/ijs.0.022178-0.

Lee, J.C., Kim, S.G., and Whang, K.S. (2015). *Sphingobium subterraneum* sp. nov., isolated from ground water. *J. Hazard Mater.* 65, 393-398. doi: 10.1099/ijs.0.069708-0.

Lee, M., Ten, L.N., Woo, S.G., and Park, J. (2011). *Agromyces soli* sp. nov., isolated from farm soil. *Int. J. Syst. Evol. Microbiol.* 61, 1286-1292. doi: 10.1099/ijs.0.021568-0.

Li, Y., Bian, D.R., Chang, J.P., Guo, L.M., and Yang, X.Q. (2020). *Sphingomonas populi* sp. nov., isolated from bark of Populus × euramericana. *Int. J. Syst. Evol. Microbiol.* 70, 897-901. doi: 10.1099/ijsem.0.003841.

Lu, C.Y., Dong, L., Li, S., Lian, W.H., Lin, Z.L., Zheng, Z.H., et al. (2023). *Salinibacterium sedimenticola* sp. nov., isolated from Tidal Flat Sediment. *Curr. Microbiol.* 80, 142. doi: 10.1007/s00284-023-03243-x.

Revesz, F., Toth, E.M., Kriszt, B., Boka, K., Benedek, T., Sarkany, O., et al. (2018). *Sphingobium aquiterrae* sp. nov., a toluene, meta- and para-xylene-degrading bacterium isolated from petroleum hydrocarbon-contaminated groundwater. *Int. J. Syst. Evol. Microbiol.* 68, 2807-2812. doi: 10.1099/ijsem.0.002898.

Saxena, A., Anand, S., Dua, A., Sangwan, N., Khan, F., and Lal, R. (2013). *Novosphingobium lindaniclasticum* sp. nov., a hexachlorocyclohexane (HCH)-degrading bacterium isolated from an HCH dumpsite. *Int. J. Syst. Evol. Microbiol.* 63, 2160-2167. doi: 10.1099/ijs.0.045443-0.

Singh, P., Kim, Y.J., Hoang, V.A., Farh Mel, A., and Yang, D.C. (2015). *Sphingomonas panacis* sp. nov., isolated from rhizosphere of rusty ginseng. *Anton. Leeuw. Int. J. G.* 108, 711-720. doi: 10.1007/s10482-015-0527-y.

Suzuki, K., Sasaki, J., Uramoto, M., Nakase, T., and Komagata, K. (1996). *Agromyces mediolanus* sp. nov., nom. rev., comb. nov., a species for "*Corynebacterium mediolanum*" Mamoli 1939 and for some aniline-assimilating bacteria which contain 2,4-diaminobutyric acid in the cell wall peptidoglycan. *Int. J. Syst. Bacteriol.* 46, 88-93. doi: 10.1099/00207713-46-1-88.

Zhang, D.C., Liu, H.C., Xin, Y.H., Yu, Y., Zhou, P.J., and Zhou, Y.G. (2008). *Salinibacterium xinjiangense* sp. nov., a psychrophilic bacterium isolated from the China No. 1 glacier. *Int. J. Syst. Evol. Microbiol.* 58, 2739-2742. doi: 10.1099/ijs.0.65802-0.

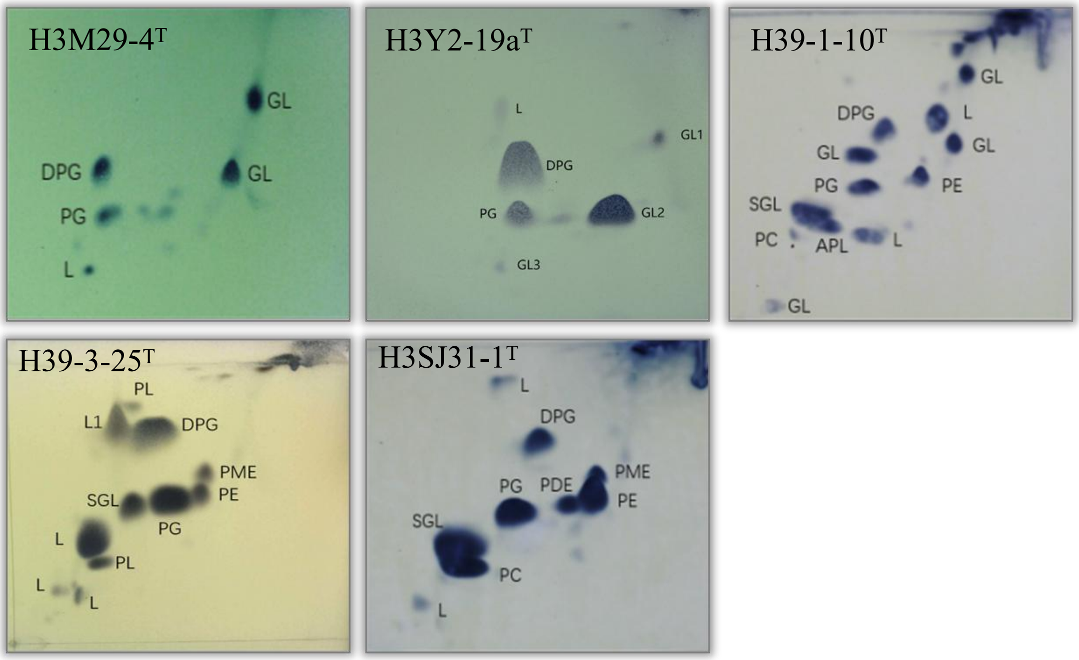

FIGURE S1 Polar lipid profiles after separation by two-dimensional thin layer chromatography of the five strains. DPG, diphosphatidylglycerol; PG, phosphatidylglycerol; PE, phosphatidylethanolamine; PME, phosphatidylmethylethanolamine; APL, unknown aminophospholipid; PL, phospholipid; GL, glycolipid; AL, aminolipid; L, unknown lipid; PC, phosphatidylcholine; PI, phosphatidylinositols; PME, phosphatidylmonomethylethanolamine, SGL, sphingoglycolipid, PDE, phosphatidyldimethylethanolamine.

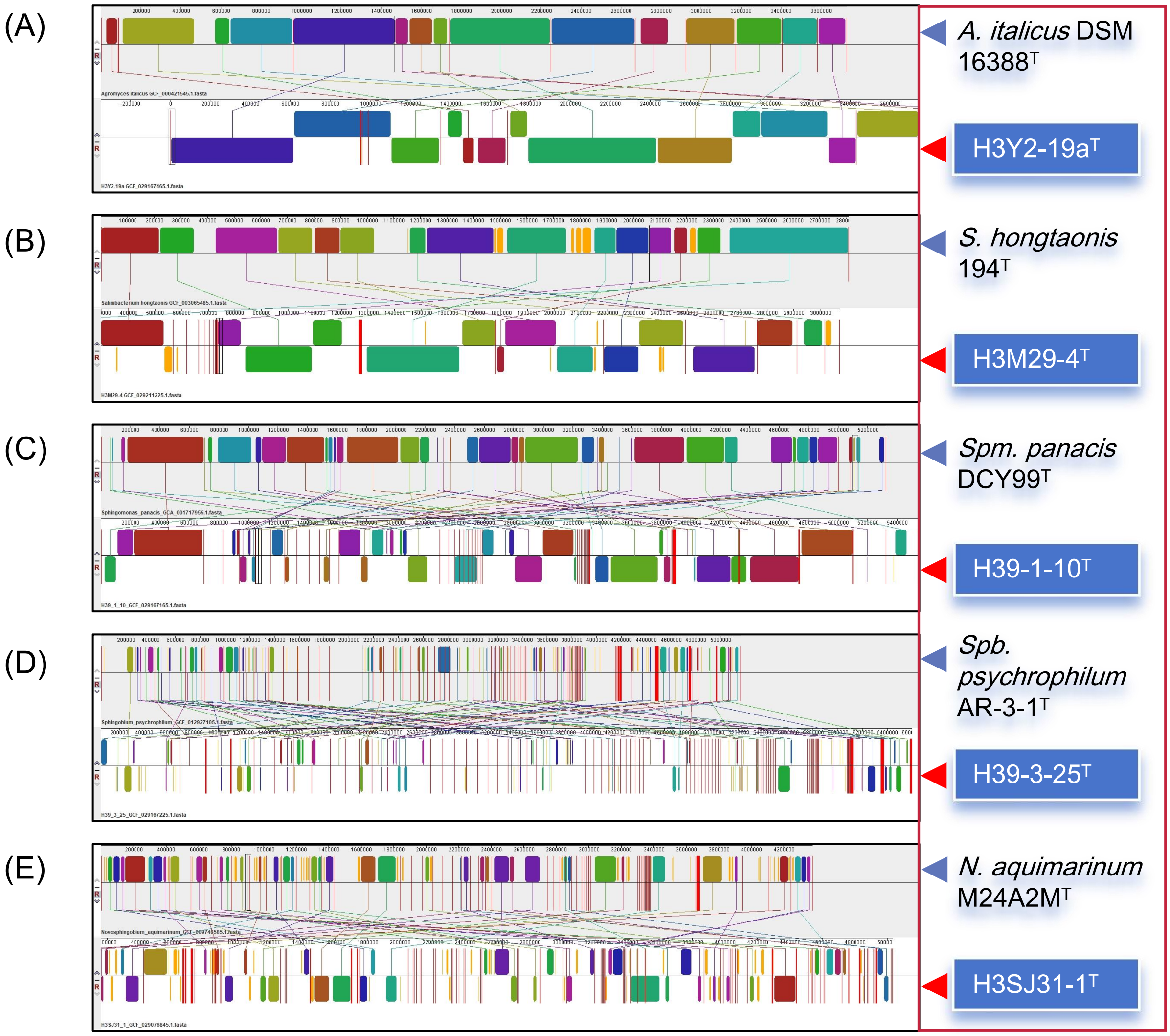

FIGURE S2 Syntenic blocks of DNA sequnce shared between the 5 strains and their nearest phylogenomic type strain neighbors. The comparative genome analysis were carried out using Mauve software. (A), syntenic blocks shared by H3Y2-19a^T and *Agromyces italicus* DSM 16388^T ; (B), by H3M29-4^T and *Salinibacterium hongtaonis* 194^T; (C), by H39-1-10^T and *Sphingomonas panacis* DCY99^T; (D), by H39-3-25^T and *Sphingobium psychrophilum* AR-3-1^T; (E), by H3SJ31-1^T and *Novosphingobium aquimarinum* M24A2M^T. The syntenic blocks upper the center line represent forward synteny, and syntenic blocks under the center line represent reverse synteny.

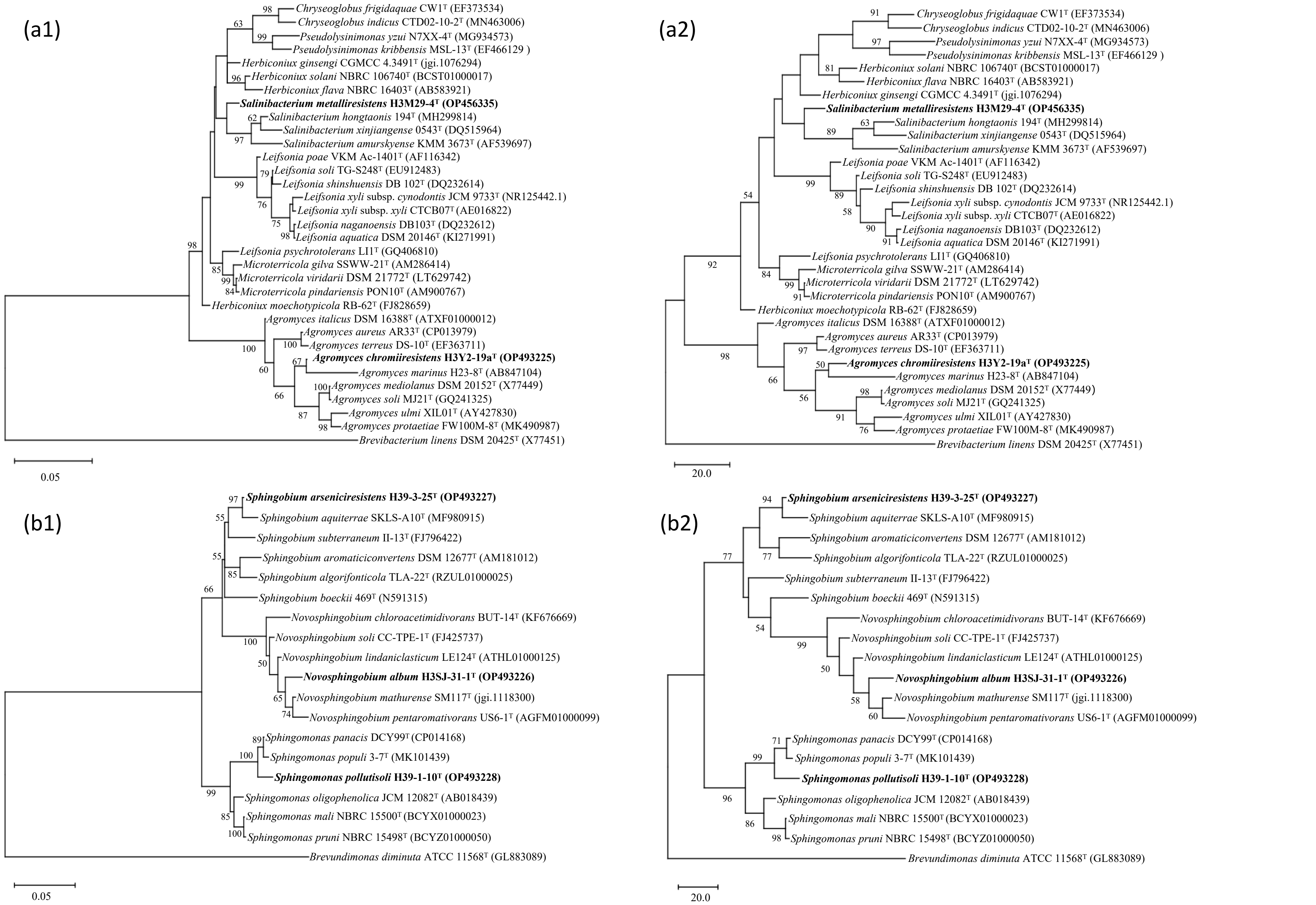

FIGURE S3 Maximum-likelihood (a1, b1) and maximum parsimony (a2, b2) phylogenetic trees based on 16S rRNA gene sequences shows the relationship between 5 strains and closely related microorganisms. Bootstrap percentages (>50%) based on 1,000 replicates are shown at the nodes. GenBank accession numbers are given in parentheses. *Brevibacterium linens* DSM 20425^{T} (X77451) was used as outgroup for (a1) and (a2). Bar, 0.05 substitutions per nucleotide position. *Brevundimonas diminuta* ATCC 11568^{T} (GL883089) was used as an outgroup for (b1) and (b2). Bar, 20.0 substitutions per nucleotide position
